# The draft genome sequence of a desert tree *Populus pruinosa*

**DOI:** 10.1093/gigascience/gix075

**Published:** 2017-08-08

**Authors:** Wenlu Yang, Kun Wang, Jian Zhang, Jianchao Ma, Jianquan Liu, Tao Ma

**Affiliations:** 1 MOE Key Laboratory for Bio-resources and Eco-environment, College of Life Science, Sichuan University, No. 24 South Section 1, Yihuan Road, 610065 Chengdu, China; 2 State Key Laboratory of Grassland Agro-Ecosystem, College of Life Science, Lanzhou University, Lanzhou, China

**Keywords:** *Populus pruinosa*, Illumina sequencing, genome assembly, annotation

## Abstract

*Populus pruinosa* is a large tree that grows in deserts and shows distinct differences in both morphology and adaptation compared to its sister species, *P. euphratica*. Here we present a draft genome sequence for *P. pruinosa* and examine genomic variations between the 2 species. A total of 60 Gb of clean reads from whole-genome sequencing of a *P. pruinosa* individual were generated using the Illumina HiSeq2000 platform. The assembled genome is 479.3 Mb in length, with an N50 contig size of 14.0 kb and a scaffold size of 698.5 kb; 45.47% of the genome is composed of repetitive elements. We predicted 35 131 protein-coding genes, of which 88.06% were functionally annotated. Gene family clustering revealed 224 unique and 640 expanded gene families in the *P. pruinosa* genome. Further evolutionary analysis identified numerous genes with elevated values for pairwise genetic differentiation between *P. pruinosa* and *P. euphratica*. We provide the genome sequence and gene annotation for *P. pruinosa*. A large number of genetic variations were recovered by comparison of the genomes between *P. pruinosa* and *P. euphratica*. These variations will provide a valuable resource for studying the genetic bases for the phenotypic and adaptive divergence of the 2 sister species.

## Background

Poplars (*Populus* spp.) are widely distributed and cultivated, and they have both economic and ecological importance. Many resequencing-based studies have been conducted to identify genetic variations responsible for their phenotypic and adaptive diversity observed in nature [1–4]. However, comparative studies based on *de novo* genome assemblies are still in their infancy since presently only 2 reference genomes are available for poplar species, namely *P. trichocarpa* (Torr. and Gray) [5] and *P. euphratica* Oliv. [6]. Further development of genome resources will offer a unique opportunity for comparative genomics and evolutionary studies within this tree genus. *P. pruinosa* Schrenk, the sister species of *P. euphratica* [7], is a large tree distributed in the deserts of western China and adjacent regions [8]. These 2 species are morphologically well differentiated. The leaves of *P. pruinosa* are ovate or kidney shaped with thick hairs, whereas *P. euphratica* has glabrous leaves with heteroblastic development. Although both species are well adapted to extreme desert environments, they grow in distinct desert habitats: *P. pruinosa* is distributed in deserts where there is highly saline underground water close to the surface, while *P. euphratica* occurs in dry deserts in which the water is deep underground and less saline [8–10]. Previous comparisons of the transcriptomes of these 2 sister species suggest that they may have developed enough genetic divergence to make it possible for them to adapt to these distinct desert habitats [9, 10]. Genomic resources and comparative genomic analysis of these 2 species would accelerate our understanding of the processes of genomic evolution underlying their phenotypic and adaptive divergence. Here we report a draft genome assembly for *P. pruinosa* and present an initial comparative genomics analysis of *P. pruinosa* and *P. euphratica*. We recovered a large number of genetic variations, including a high level of heterozygosity, several genes that had undergone rapid evolution, and numerous gene families that were unique and expanded in *P. pruinosa* genome.

## Data description

### Samples and sequencing

High-quality genomic DNA was extracted from the leaf tissues of a single *P. pruinosa* tree (NCBI Taxonomy ID: 492 479) collected in Xinjiang, China, using the cetyl trimethylammonium bromide (CTAB) method [11]. Sequencing libraries with different insert sizes were constructed according to the Illumina protocol. Briefly, for paired-end libraries with insert sizes ranging from 158 to 780 bp, DNA was fragmented, end-repaired, A-tailed, and ligated to Illumina paired-end adapters (Illumina). The ligated fragments were size-selected on agarose gel and amplified by ligation-mediated polymerase chain reaction (PCR) to produce the corresponding libraries. For mate pair libraries (2 to 20 kb), about 20–50 μg genomic DNA was fragmented using nebulization for 2 kb, or HydroShear (Covaris) for 5, 10, and 20 kb. Next, the DNA fragments were end-repaired using biotinylated nucleotide analogues and purified using the QIAquick PCR Purification Kit (Qiagen). Then the target fragments were selected on agarose gel and circularized by intramolecular ligation. Circular DNA was fragmented (Covaris), and biotinylated fragments were purified with magnetic beads (Invitrogen), end-repaired, A-tailed, and ligated to Illumina paired-end adapters, size-selected again, and purified using the QIAquick Gel Extraction kit (QIAGEN). All of the above libraries were sequenced on an Illumina HiSeq 2000 platform. For the data filtering process, we discarded reads that met either of the following criteria: (i) reads with ≥10% unidentified nucleotides; (ii) reads from paired-end libraries having more than 40% bases with Phred quality <8, and reads from mate pair libraries that contained more than 60% bases with quality <8; (iii) reads with more than 10 bp aligned to the adapter sequence, allowing <4 bp mismatch; (iv) reads from paired-end libraries that overlapped ≥10 bp with the corresponding paired end. We also corrected the reads containing sequencing errors and removed the duplicates introduced by PCR amplification in paired reads using Lighter v. 1.0.7 [12] and FastUniq v. 1.1 (FastUniq, RRID:SCR_000682) [13], respectively. Finally, ∼60 Gb of clean data (Additional file 1: Table S1) were obtained for the *de novo* assembly of the *P. pruinosa* genome.

Clean reads obtained from paired-end libraries were subjected to 17-mer frequency distribution analysis with KmerFreq_AR [14]. Analysis parameters were set at -k 17 -t 10 -q 33, and the final result was plotted as a frequency graph (Additional file 1: Figure S1). Two distinctive peaks observed from the distribution curve demonstrated the high heterozygosity of the *P. pruinosa* genome. To prevent the deviation of *k*-mer-based methods on the estimation of genome size, we determined the genome size of *P. pruinosa* with flow cytometry, using *Vigna radiata* as reference standard and propidium iodide as the stain. Our flow cytometry analysis showed that the genome size of *P. pruinosa* was approximately 590 Mb (Additional file 1: Figure S2).

In addition, 3 tissues (leaf, phloem, and xylem) of a 2-year-old *P. pruinosa* plant collected from Tarim Basin desert in Xinjiang were harvested and flash frozen in liquid nitrogen, and then the RNA were extracted using the CTAB method [11, 15]. RNA-seq libraries were constructed using the NEB Next Ultra Directional RNA Library Prep Kit for Illumina (NEB, Ispawich, USA) according to the manufacturer's instructions, and libraries were sequenced using an Illumina HiSeq 2500 platform with a read length of 2 × 125 bp. More than 38 million paired-end reads were generated for each sample (Additional file 1: Table S2). We next assembled these RNA-seq reads using Trinity v. 2.1.1 (Trinity, RRID:SCR_013048) [16] with the default parameters and reduced the redundancy of transcript sequences (>95% similarity) using CD-Hit v. 4.6.1 (CD-HIT, RRID:SCR_007105) [17]. The software TransDecoder v. 2.1.0 [18] was used to identify candidate coding regions within these transcript sequences. Finally, a total of 111 538 unigenes were obtained for subsequent evaluation of gene space completeness of our genome assembly and transcriptome-based gene prediction.

### Genome assembly

The *P. pruinosa* genome was *de novo* assembled using Platanus v. 1.2.1 (Platanus, RRID:SCR_015531) [19] with a default parameter of -k 32, which is optimized for highly heterozygous diploid genomes. Briefly, the clean reads derived from paired-end libraries were first split into *k*-mers to construct *de Bruijn* graphs and then merged into distinct contigs based on overlap information. All reads from paired-end and mate pair libraries were then aligned against the contigs, and the paired relationships were used to link contigs into scaffolds. Finally, the intra-scaffold gaps were closed by local assembly implemented in GapCloser v. 1.12 (GapCloser, RRID:SCR_015026) [20] using the paired-end reads for which 1 end uniquely mapped to a contig but the other end was located within a gap. After discarding the scaffolds smaller than 200 bp, we yielded a draft assembly with a total length of 479.3 Mb (Table 1), which covers 85% of the predicted genome size of *P. pruinosa*. The contig and scaffold N50 sizes were 14.0 kb and 698.5 kb respectively, while the unclosed gap regions represent 6.08% of the assembly (Additional file 1: Table S3). The distribution of the average guanylic and cytidylic acid (GC) content of the *P. pruinosa* genome (mean = 31.8%) is similar to that of the *P. euphratica* genome (32.1%) [6] and the *P. trichocarpa* genome (33.6%) (Additional file 1: Figure S3) [5].

**Table 1:** Summary of genome assembly and annotation of *P. pruinosa*

Genome assembly	
Estimate of genome size	590 Mb
GC content	31.80%
Contigs	
N50 size	14 011 bp
Longest	197 623 bp
Total number	170 219
Total size	450 157 195 bp
Scaffolds	
N50 size	698 525 bp
Longest	10 688 665 bp
Total number	78 960
Total length	479 307 600 bp
Genome annotation	
Transposable elements	
LTR	142 923 156 bp (29.82%)
LINE	4 956 260 bp (1.03%)
DNA	20 990 612 bp (4.38%)
Total	213 236 753 bp (45.47%)
Protein coding genes	
Total number	35 131
Mean transcript length	3703.4 bp
Mean coding sequence length	1224.38 bp
Mean exon length	226.27 bp
Mean intron length	561.98 bp
Functional annotation	
GO	22 361 (63.64%)
KEGG	11 746 (33.43%)
Total	30 938 (88.06%)

To evaluate the completeness of this assembly, we first examined the coverage of highly conserved genes using BUSCO (BUSCO, RRID:SCR_015008) [21]. The result showed that 922 out of the 956 conserved genes (96.44%) could be found in our assembly, of which 699 were single and 223 were duplicated, and only 10 (1.05%) genes had fragmented matches (Additional file 1: Table S4). These coverage values were comparable to estimates for the *P. euphratica* and *P. trichocarpa* genomes. Furthermore, the 111 538 *P. pruinosa* unigenes obtained in this study and the protein-coding genes predicted in the *P. euphratica* and *P. trichocarpa* genomes [5, 6] were aligned to our genome assembly using the BLAT algorithm with default parameters. Statistical analysis was done at different levels of percentage of sequence homology and percentage of coverage. The results showed that our assembly covered approximately 90% of the *P. pruinosa* unigenes and 99% and 98% of the protein-coding genes in *P. euphratica* and *P. trichocarpa*, respectively (Additional file 1: Table S5). Finally, we applied the Feature-Response Curves (FRC) v. 1.3.0 method [22] to evaluate the trade-off between the contiguity and correctness of our assembly. This method is based on a prediction of assembly correctness by identifying each *de novo* assembled scaffold, “features” representing potential errors, or complications during the assembly process. Evaluation using the FRC method and our genome sequencing reads indicated that the *P. pruinosa* genome assembly certainly generated a better FRCurve than the other 3 Salicaceae species assemblies (Additional file 1: Figure S4), suggesting that the continuity of our assembly is acceptable. In summary, all of these statistics revealed that our draft genome sequence has high contiguity, accuracy, and, more importantly, a high degree of gene space completeness for effective gene detection.

We mapped the clean reads from the paired-end libraries to the *P. pruinosa* genome using the Burrows-Wheeler Aligner v. 0.7.12-r1044 (BWA, RRID:SCR_010910) [23] and found that the sequencing depth for 95.3% of the assembly was more than 20-fold (Additional file 1: Figure S5), ensuring a high level of accuracy at the nucleotide level. We also performed variant calling using the Genome Analysis Toolkit v. 3.5 (GATK, RRID:SCR_001876) [24]. A total of 3.11 million heterozygous single nucleotide variants (SNVs) were obtained after strict quality control and filtering, which revealed that the heterozygosity level of the *P. pruinosa* genome was approximately 0.80%.

### Repeat annotation

Repetitive sequences and transposable elements (TEs) in the *P. pruinosa* genome were identified using a combination of *de novo* and homology-based approaches at both the DNA and protein levels. Initially, we built a *de novo* repeat library for *P. pruinosa* using RepeatModeler v. 1.0.8 (RepeatModeler, RRID:SCR_015027) [25] with default parameters. For identification and classification of transposable elements at the DNA level, RepeatMasker (RepeatMasker, RRID:SCR_012954) [25] was applied to map our assembly against both the databases that we had built and the known Repbase [26] transposable element (TE) library. Next we executed RepeatProteinMask [25] using a WU-BLASTX search against the TE protein database to further identify repeats at the protein level. In addition, we annotated tandem repeats using the software Tandem Repeat Finder (TRF v. 4.07b) [27]. In total, the combined non-redundant results showed that approximately 45% of the *P. pruinosa* genome assembly is composed of repetitive elements (Additional file 1: Table S6), a value similar to that of the *P. euphratica* genome (44%). Long terminal repeats (LTRs) were the most abundant repeat class, accounting for 67.03% of repetitive sequences, representing 29.82% of the genome (Additional file 1: Table S7).

### Gene annotation

We conducted the gene annotation in the *P. pruinosa* genome by combining homology-based, *de novo*, and transcriptome-based methods. For homology-based prediction, protein sequences from 6 sequenced plants (*P. euphratica* [6], *P. trichocarpa* [5], *Ricinus communis* [28], *Arabidopsis thaliana* [29], *Carica papaya* [30], and *Eucalyptus grandis* [31]) were aligned to the *P. pruinosa* genome using TBLASTN v. 2.2.26 [32]. The homologous genome sequences were then aligned against the matching proteins using GeneWise v. 2.4.1 (GeneWise, RRID:SCR_015054) [33] to obtain accurate spliced alignments. For *de novo* prediction, we performed Augustus v. 3.2.1 (Augustus: Gene Prediction, RRID:SCR_008417) [34] and GenScan (GENSCAN, RRID:SCR_012902) [35] analysis on the repeat-masked genome with parameters trained from *P. pruinosa* and *A. thaliana*. The resultant data sets were filtered with the removal of partial sequences and genes, with coding lengths of less than 100 bp. For the transcriptome-based approach, the 111 538 *P. pruinosa* transcripts obtained above were aligned to the *P. pruinosa* genome and further assembled using the Program to Assemble Spliced Alignments v. 2.0.2 (PASA, RRID:SCR_014656) [36] to detect likely protein coding regions. Finally, we combined the gene annotation results from all homology-based, *de novo*, and transcriptome-based predictions using EVM v. 1.1.1 (EVidenceModeler, RRID:SCR_014659) [37] to produce a consensus protein-coding gene set.

In sum, the *P. pruinosa* genome contains 35 131 protein-coding genes with an average coding sequence (CDS) length of 1224 bp (Additional file 1: Table S8). The length distributions of transcripts, coding sequences, exons, and introns were similar in *P. euphratica* and in *P. trichocarpa* (Additional file 1: Figure S6). Functional annotation was performed based on comparisons with the SwissProt, TrEMBL [38], InterPro [39], and KEGG [40] protein databases. Gene ontology (GO) [41] IDs for each gene were assigned by the Blast2GO pipeline (Blast2GO, RRID:SCR_005828) [42] based on NCBI databases. Overall, 75.43% of the protein-coding genes had conserved protein domains, and 63.64% could be classified by GO terms (Additional file 1: Table S9).

### Evolutionary analysis

**Figure 1:**
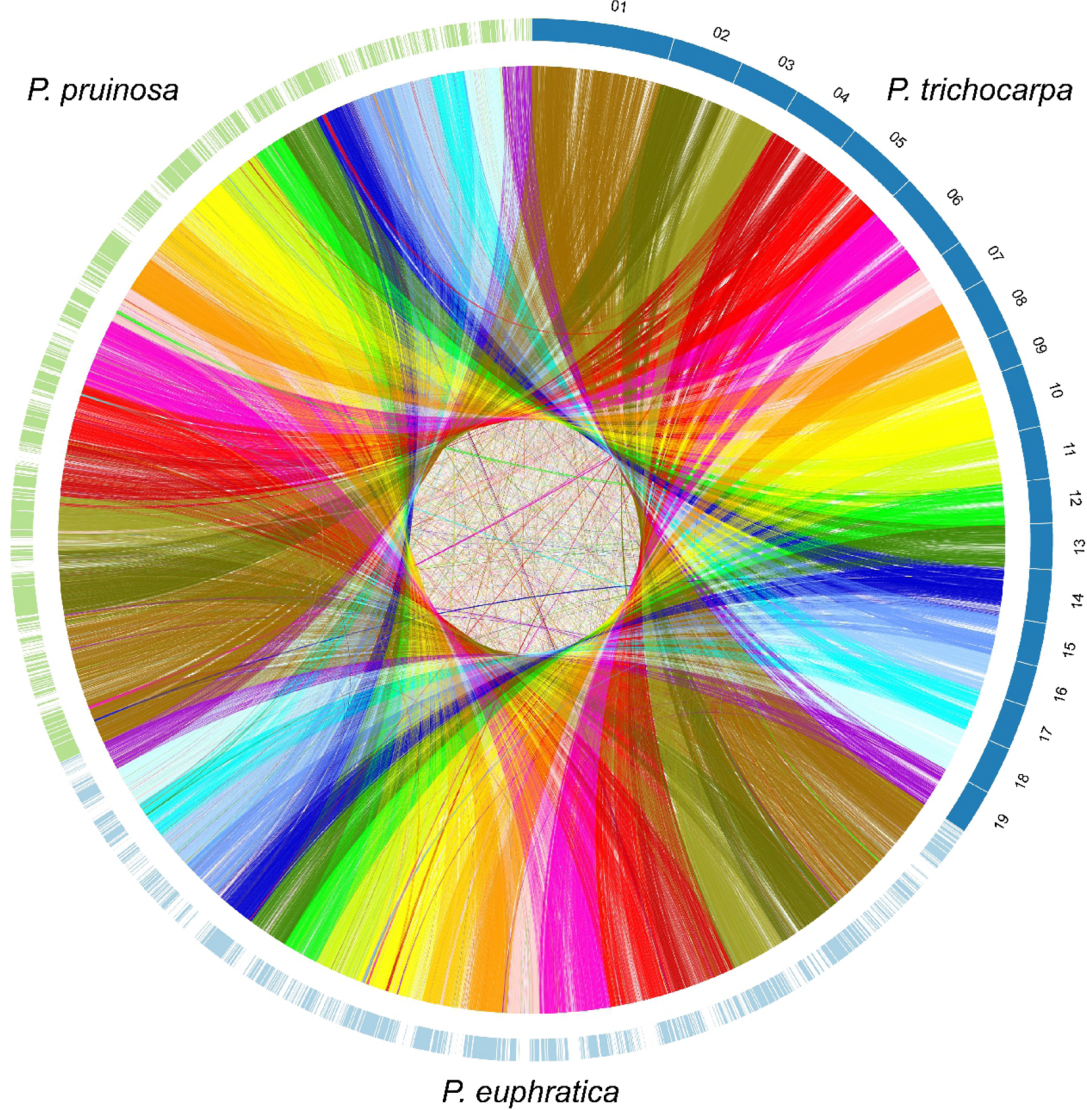
Synteny relationship of *P. pruinosa, P. euphratica*, and *P. trichocarpa*.

Blocks syntenic between *P. pruinosa* and *P. euphratica* were determined by the software MCScanX [43]; at least 5 genes were required to call synteny. The blocks identified occupy the majority of the genome assemblies of *P. pruinosa* (290 Mb, 66% of the assembly; 29 015 genes, 83% of the predicted gene models) and *P. euphratica* (293 Mb, 59%; 27 804 genes, 81%) (Additional file 1: Table S10), suggesting that there is extensive macrosynteny between these 2 species. This overall high level of synteny was also confirmed by whole-genome alignment using the program “LAST” (Fig. 1) [44]. A total of 15 695 high-confidence 1:1 orthologous genes were identified in these syntenic blocks. We estimated and plotted the nucleotide synonymous substitution (Ks) rates for these orthologous pairs, and a peak at around 0.016 was observed (Additional file 1: Figure S7), while the divergence between duplicated genes in *P. pruinosa* and *P. euphratica* peaked around 0.272 and 0.257, respectively, indicating that the 2 species had shared common whole-genome duplication (WGD) events before they diverged from a common ancestor. Adaptive divergence at the molecular level may be reflected in an increased rate of nonsynonymous changes within genes involved in adaptation [45]. We found that the mean similarity between *P. euphratica* and *P. pruinosa* orthologous genes at the protein level is close to 97.22% (Additional file 1: Figure S8). Average synonymous (Ks) and nonsynonymous (Ka) gene divergence values were 0.04 and 0.017, respectively. The genes that showed elevated pairwise genetic differentiation were enriched mainly in “metal ion transport,” “regulation of gene expression,” “response to stimulus,” “antiporter activity,” “heat shock protein binding,” and “oxidoreductase activity” (Additional file 1: Table S11), indicating that these functions had undergone rapid evolution (caused by adaptive divergence and/or relaxed selection) between *P. pruinosa* and *P. euphratica*.

Gene family clustering analysis was performed using OrthoMCL v. 3.1 (OrthoMCL: Ortholog Groups of Protein Sequences, RRID:SCR_007839) [46] on all the protein-coding genes of *P. pruinosa* and 10 additional species (*P. euphratica, P. trichocarpa, Salix suchowensis, Ricinus communis, Arabidopsis thaliana, Carica papaya, Fragaria vesca, Cucumis sativus, Eucalyptus Grandis*, and *Vitis vinifera*). Of the 35 131 protein-coding genes in *P. pruinosa*, 28 773 (81.9%) could be classified into a total of 17 592 families, with 224 clusters comprising 662 genes specific to *P. pruinosa* (Additional file 1: Table S12). We identified a total of 7020 *P. pruinosa*–specific genes, of which 3639 (51.8%) were supported by gene expression data (RPKM > 0.5) and/or functional annotation (Additional file 1: Table S13), indicating that there is a large number of species-specific genes even though the genomes of *P. pruinosa* and *P. euphratica* are closely related to each other. Further analysis revealed that these *P. pruinosa*–specific genes were primarily enriched in “transcription factor activity,” “transporter activity,” “response to salt stress,” and “oxidoreductase activity” (Additional file 1: Table S14).

In addition, we identified a total of 1354 single-copy gene families across the 11 plant genomes. Alignments were generated for each family with MUSCLE v. 3.8.31 (MUSCLE, RRID:SCR_011812) [47], and low-quality regions of the alignments were identified and trimmed with Gblocks v. 0.91b [48, 49] using default parameters. The individual trimmed protein-coding alignments were concatenated into 1 “supergene” for each species in order to construct a phylogenetic tree using RAxML v. 8.2.8 (RaxML, RRID:SCR_006086) (Additional file 1: Figure S9) [50]. Then MCMCTree v. 4.9 [51] was applied to estimate the divergence time based on the phylogenetic relationships, using fossil calibration times for divergence between *A. thaliana* and *C. papaya* (54–90 million years ago [Mya]), *A. thaliana* and *R. communis* (95–109 Mya), and *V. vinifera* and *A. thaliana* (106–119 Mya), which were obtained from the TimeTree database [52]. The divergence time between *P. pruinosa* and *P. euphratica* was estimated to be 3.0 Mya (1.6–5.0 Mya) (Additional file 1: Figure S10). Last, we applied the Computational Analysis of gene Family Evolution (CAFÉ) v. 3.1 [53] program to examine gene family evolution across entire genomes. The results showed that 640 gene families related to “glucosyltransferase activity,” “ADP binding,” “cation channel activity,” “cell differentiation” and “oxidoreductase activity” were substantially expanded in *P. pruinosa* compared to other plant species (Additional file 1: Table S15 and Figure S11).

In summary, we present here the sequencing, assembly, and annotation of the genome *P. pruinosa* and compare it with that of its sister species *P. euphratica*. Although a high level of overall similarity was observed between the 2 genomes, our evolutionary analyses identified a large number of genes showing signs of rapid divergence and numerous species-specific genes, which may have resulted from rapid habitat adaptation and natural selection during speciation of the 2 species. However, population genomic analyses will be needed in order to examine whether these variations are widely fixed across all populations of each species. In addition, functional tests should be performed to explore the roles that variations play in both morphological and ecological divergence.

## Availability of supporting data

The sequencing reads from each sequencing library have been deposited at NCBI with the Project ID PRJNA353148 and Sample ID SAMN06011208. The assembly and annotation of the *P. pruinosa* genome, the assembly pipeline, and commands used in this work are available in the *GigaScience* database, *Giga*DB [54]. All supplementary figures and tables are provided in Additional file 1.

## Additional files

Table S1: Summary of clean reads after the raw reads from the Illumina platform had been filtered using Lighter and FastUniq.

Table S2: Statistics for *P. pruinosa* RNA-seq data.

Table S3: Statistics for the final assembly of the *P. pruinosa* genome.

Table S4: Summary of BUSCO analysis.

Table S5: Evaluation of gene space completeness for the *P. pruinosa* genome.

Table S6: Prediction of repetitive elements in the *P. pruinosa* genome.

Table S7: Classification of repetitive elements in the *P. pruinosa* genome.

Table S8: Statistics of predicted protein-coding genes in the *P. pruinosa* genome.

Table S9: Functional annotation of predicted genes for *P. pruinosa.*

Table S10: Summary of syntenic blocks between *P. pruinosa* and *P. euphratica* identified using MCScanX.

Table S11: Top 10 GO categories (biological process and molecular function) displaying the highest Ka/Ks ratios between *P. pruinosa* and *P. euphratica*.

Table S12: Summary of gene family clustering.

Table S13: Analysis of *P. pruinosa* species-specific genes.

Table S14: GO enrichment analysis of species-specific genes in the *P. pruinosa* genome.

Table S15: GO enrichment analysis of expanded gene families in the *P. pruinosa* genome.

Figure S1: 17-mer analysis for *P. pruinosa* genome based on clean reads from paired-end libraries.

Figure S2: Flow cytometry estimate of the *P. pruinosa* genome size compared to the reference standard of *Vigna radiate* (543 Mb).

Figure S3: GC content distribution for the genomes of *P. pruinosa* and related poplar species, established by 500 bp non-overlapping sliding windows.

Figure S4: FRCurve of 4 genome assemblies.

Figure S5: Sequencing depth distribution for the *P. pruinosa* genome.

Figure S6: Comparison of mRNA length (A), CDS length (B), exon length (C), intron length (D), and exon number per gene (E) in *P. pruinosa* and related poplar species.

Figure S7: Genome duplication in *Populus* genomes as revealed by Ks analyses.

Figure S8: Distribution of Ka, Ks, Ka/Ks, and protein similarity in 1:1 *P. pruinosa–P. euphratica* orthologs within syntenic blocks.

Figure S9: Phylogenetic relationships of *P. pruinosa* and 10 other plant species.

Figure S10: Estimation of divergence time between *P. pruinosa* and *P. euphratica* using phylogenetic analysis.

Figure S11: Dynamic evolution of orthologous gene families.

## Abbreviations

bp: base pair; CDS: coding sequence; Gb: giga base; kb: kilo base; Mb: mega base; SRA: Sequence Read Archive; TE: transposable element.

## Competing interests

The authors declare that they have no competing interests.

## Funding

This project was supported by the National Key Research and Development Program of China (2016YFD0600101), the National Key Project for Basic Research (2012CB114504), the National Natural Science Foundation of China (31561123001 and 31500502), and the Fundamental Research Funds for the Central Universities.

## Supplementary Material

GIGA-D-16-00147_Original-Submission.pdf

GIGA-D-16-00147_Revision-1.pdf

GIGA-D-16-00147_Revision-2.pdf

GIGA-D-16-00147_Revision-3.pdf

Response-to-Reviewer-Comments_Original-Submission.pdf

Response-to-Reviewer-Comments_Revision-1.pdf

Response-to-Reviewer-Comments_Revision-2.pdf

Reviewer-1-Report-(Original-Submission).pdf

Reviewer-1-Report-(Revision-1).pdf

Reviewer-2-Report-(Original-Submission).pdf

Reviewer-2-Report-(Revision-1).pdf

Reviewer-3-Report-(Original-Submission).pdf

Additional Tables
