# Supplementary material for: The draft genome sequence of a desert tree Populus pruinosa
Source: Gigascience. 2017 Aug 8;6(9):1–7. doi: 10.1093/gigascience/gix075 (PMC5603765; doi:10.1093/gigascience/gix075)
Supplement: GIGA-D-16-00147_Original-Submission.pdf [file gix075_GIGA-D-16-00147_Original-Submission.pdf]

Manuscript Click here to download Manuscript PprGenome-V6.docx

The draft genome sequence of a desert tree *Populus pruinosa*

Wenlu Yang[1], Jian Zhang[2], Jianchao Ma[2], Jianquan Liu[1,2], Tao Ma[1*]

[1]MOE Key Laboratory for Bio-resources and Eco-environment, College of Life Science, Sichuan University, Chengdu, China

[2]State Key Laboratory of Grassland Agro-Ecosystem, College of Life Science, Lanzhou University, Lanzhou, China

*Correspondence should be addressed to T. M. (matao.yz@gmail.com)

Abstract

Background

Populus pruinosa is a large tree that grows in deserts and shows distinct differences in both morphology and adaptation from those of the sister species, *P. euphratica*. Here we present a draft genome sequence for *P. pruinosa* and examine genomic variations between the two species.

Findings

A total of 60 Gb of qualified reads from whole-genome sequencing of a *P. pruinosa* individual were generated using the Illumina HiSeq2000 platform. The assembled genome is 479.3 Mb in length, with an N50 contig size of 14.0 kb and a scaffold size of 698.5 kb. 45% of the genome is composed of repetitive elements. We predicted 35,139 protein-coding genes, of which 88% were functionally annotated. Gene family clustering revealed 209 unique and 613 expanded gene families in the *P. pruinosa* genome. Further evolutionary analysis identified numerous genes with elevated values for pairwise genetic differentiation between *P. pruinosa* and *P. euphratica*, and these genes are particularly enriched in functions related to the different adaptations of the two species to their specialized desert habitats.

Conclusions

The large number of genetic variations recovered here suggest that it will be necessary to carry out examinations of the *Populus* pan-genomes at both the species and the population level in the future. These variations also provide a valuable resource for studying the genetic bases for the phenotypic and adaptive divergence of the two sister species.

Keywords

Populus pruinosa, Illumina sequencing, Genome assembly, Comparative genomics

Background

Poplars (*Populus* spp.) are widely distributed and cultivated, and they have both economic and ecological importance. Despite their remarkable diversity, relatively little is known about the evolutionary genomics of this tree genus. While reference genomes are available for two poplar species, namely *P. trichocarpa* [1] and *P. euphratica* [2], they are still insufficient to capture the entire range of genomic variation responsible for the phenotypic and adaptive diversity observed among poplars in nature. *P. pruinosa*, the sister species of *P. euphratica* [3], is a large tree distributed in the deserts of western China and adjacent regions [4]. These two species are morphologically well differentiated. The leaves of *P. pruinosa* are ovate or kidney-shaped with thick hairs, whereas *P. euphratica* has glabrous leaves with heteroblastic development. Although both species are well adapted to extreme desert environments, they grow in the specialized desert habitats: *P. pruinosa* is distributed in deserts where there is highly saline underground water close to the surface, while *P. euphratica* occurs in dry deserts in which the water is deep underground and less saline [4-6]. Previous comparisons of the transcriptomes of these two sister species suggest that they may have developed enough genetic divergence to make it possible for them to adapt to these specialized desert habitats [5, 6]. Genomic resources and comparative genomic analysis of these two species would accelerate our understanding of the processes of genomic evolution underlying their phenotypic and adaptive divergence. Here we report a draft genome assembly for *P. pruinosa* and present an initial comparative genomics analysis of *P. pruinosa* and *P. euphratica*. We recovered an unexpectedly large number of genetic variations between these two sister tree species.

Data description

Samples and Sequencing

Genomic DNA was extracted from the leaf tissues of a single *P. pruinosa* tree (NCBI Taxonomy ID: 492479) collected in Xinjiang, China. Sequencing libraries with different insert sizes were constructed according to the Illumina protocol. For small-insert (158, 483 and 780 bp) libraries, DNA was fragmented, end repaired, ligated to

Illumina paired-end adapters and purified by PCR amplification. For large-insert (2 to 20 kb) mate-paired libraries, the genomic DNA was circularized, fragmented, purified as biotinylated DNA and ligated to adapters. All of the above libraries were sequenced on an Illumina HiSeq 2000 platform. The acquired raw reads were processed by removing low-quality reads, adapter sequences and possible contaminated reads using Lighter [7] and FastUniq [8]. Finally, about 60 Gb of clean data (Additional file 1: Table S1) were obtained for the *de novo* assembly of the *P. pruinosa* genome.

Qualified reads obtained from small-insert libraries were subjected to 17-mer frequency distribution analysis with KmerFreq_AR [9]. Analysis parameters were set at -k 17 -t 10 -q 33, and the final result was plotted as a frequency graph (Additional file 1: Figure S1), which shows two distinctive peaks: (i) the first peak demonstrates the high level of heterozygosity of the *P. pruinosa* genome; and (ii) the second peak provides a peak depth for the estimation of genome size. Using the formula genome size = *k*-mer_Number/Peak_Depth, the size of the *P. pruinosa* genome was estimated to be approximately 439 Mb (Additional file 1: Table S2).

Genome assembly

The *P. pruinosa* genome was *de novo* assembled by Platanus [10], which is optimized for highly heterozygous diploid genomes. Briefly, the qualified reads derived from small-insert libraries were firstly split into *k*-mers to construct *de Bruijn* graphs and merged into distinct contigs based on overlap information. All reads from small- and large-insert libraries were then aligned against the contigs and the paired-end relationships were used to link contigs into scaffolds. Finally, the intra-scaffold gaps were closed by local assembly implemented in GapCloser [11] using the paired-end reads for which one end uniquely mapped to a contig but the other end was located within a gap. This yielded a draft *P. pruinosa* genome of about 479.3Mb, with contig and scaffold N50 sizes of 14.0 kb and 698.5 kb respectively (Additional file 1: Table S3). The distribution of the average GC content of the *P. pruinosa* genome (mean: 31.8%) is similar to that for the *P. euphratica* genome [2] (32.1%) and the *P. trichocarpa* genome [1] (33.6%) (Additional file 1: Figure S2).

To evaluate the completeness of this assembly, we examined the coverage of highly conserved genes using CEGMA [12] and BUSCO [13]. The results showed that our assembly captured 95.97% (238 of 248) of the core CEGMA genes, with 91.94% (228) of them being complete (Additional file 1: Table S4). 96.44% of the 956 conserved genes were recovered in the BUSCO analysis, and of these 699 were single and 223 were duplicated (Additional file 1: Table S5). These coverage values were comparable to estimates for the *P. euphratica* and *P. trichocarpa* genomes, indicating that the degree of gene space completeness was sufficiently high for effective gene detection in our genome assembly.

We also mapped the qualified reads from the small-insert libraries to the *P. pruinosa* genome using the Burrows-Wheeler Aligner (BWA) [14] and found that the sequencing depth for 95.3% of the assembly was more than 20-fold (Additional file 1: Figure S3), ensuring a high level of accuracy at the nucleotide level. We also performed variant calling using the Genome Analysis Toolkit (GATK) [15]. A total of 3.21 million heterozygous single nucleotide variants (SNVs) were obtained after strict quality control and filtering. This revealed that the heterozygosity level of the *P. pruinosa* genome was approximately 0.86%, which is higher than that estimated for the *P. euphratica* genome (0.49%) [2].

Repeat annotation

Repetitive sequences and transposable elements (TEs) in the *P. pruinosa* genome were identified using a combination of *de novo* and homology-based approaches at both the DNA and the protein level. Initially, we built a *de novo* repeat library for *P. pruinosa* using RepeatModeler [16] with default parameters. For identification and classification of transposable elements at the DNA level, RepeatMasker [16] was applied to map our assembly against both the databases that we had built and the known Repbase [17] transposable element (TE) library. Next we executed RepeatProteinMask [16] using a WU-BLASTX search against the TE protein database to further identify repeats at the protein level. In addition, we annotated tandem repeats using the software Tandem Repeat Finder (TRF) [18]. In total, we found that approximately 45% of the *P. pruinosa*

genome assembly is composed of repetitive elements (Additional file 1: Table S6), a value similar to that for the *P. euphratica* genome (44%). Long terminal repeats (LTRs) were the most abundant repeat class, accounting for 67.03% of repetitive sequences representing 29.82% of the genome (Additional file 1: Table S7).

Gene annotation

We combined homology-based, *de novo* and transcriptome-based methods to predict the gene content of this assembly. For homology-based prediction, protein sequences from five sequenced plants (*P. euphratica*, *P. trichocarpa*, *Ricinus communis*, *Arabidopsis thaliana* and *Carica papaya*) were aligned to the *P. pruinosa* genome using TBLASTN [19]. The resultant homologous genome sequences were then aligned against the matching proteins using GeneWise [20] to obtain accurate spliced alignments. For *de novo* prediction, we applied Augustus [21] and GenScan [22] to the repeat masked genome, and filtered out partial genes and small genes with coding length less than 100 bp. For the transcriptome-based approach, total RNAs were first extracted from leaf, root, xylem and phloem of a 2-year-old seedling and sequenced using an Illumina HiSeq 2500 platform (Additional file 1: Table S8). Then we assembled these RNA-seq reads using Trinity [23] with the default parameters and reduced the redundancy of transcript sequences (>95% similarity) using CD-Hit [24]. The software TransDecoder [25] was used to identify candidate coding regions within transcript sequences. These sequences were then aligned to the *P. pruinosa* genome and further assembled using the Program to Assemble Spliced Alignments (PASA) [26]. Finally, all the predictions obtained above were combined using EVidenceModeler (EVM) [27] to produce a consensus protein-coding gene set. In total, the *P. pruinosa* genome contains 35,139 protein-coding genes with an average CDS length of 1,224 bp (Additional file 1: Table S9). The length distributions of transcripts, coding sequences, exons and introns were similar in *P. euphratica* and in *P. trichocarpa* (Additional file 1: Figure S4). Functional annotation was performed based on comparisons with the SwissProt, TrEMBL [28], InterPro [29] and KEGG [30] protein databases. Gene Ontology (GO) [31] IDs for each gene were assigned by the Blast2GO pipeline [32]

based on NCBI databases. Overall, 62.35% of the protein-coding genes had conserved protein domains and 63.59% could be classified by GO terms (Additional file 1: Table S10).

Evolutionary analysis

Blocks syntenic between *P. pruinosa* and *P. euphratica* were determined by the software MCScanX [33], at least five genes were required to call synteny. The blocks identified occupy the majority of the genome assemblies of *P. pruinosa* (290 Mb, 66% of the assembly; 29,006 genes, 83% of the predicted gene models) and *P. euphratica* (293 Mb, 59%; 27,782 genes, 81%) (Additional file 1: Table S11), suggesting that there is extensive macrosynteny between these two species. A total of 15,719 high-confidence 1:1 orthologous genes were identified in these blocks. We estimated and plotted the nucleotide synonymous substitution (Ks) rates for these orthologous pairs, and a peak at around 0.015 was observed (Additional file 1: Figure S5), while the divergence between duplicated genes in *P. pruinosa* and *P. euphratica* peaked around 0.271 and 0.256, respectively, indicating that the two species had shared common whole genome duplication (WGD) events before they diverged from a common ancestor. Adaptive divergence at the molecular level may be reflected in an increased rate of nonsynonymous changes within genes involved in adaptation [34]. We found that the mean similarity between *P. euphratica* and *P. pruinosa* orthologous genes at the protein level is close to 97.22% (Additional file 1: Figure S6). Average synonymous (Ks) and nonsynonymous (Ka) gene divergence values were 0.04 and 0.017 respectively. The genes that showed elevated pairwise genetic differentiation were enriched mainly in 'superoxide metabolic process', 'response to freezing', 'regulation of ion transmembrane transport', 'heat shock protein binding' and 'ADP binding' terms (Additional file 1: Table S12), indicating that these functions had undergone rapid evolution and/or adaptive divergence between *P. pruinosa* and *P. euphratica*. These functional categories are probably related to the differences in the adaptations of these two species to their specialized desert habitats [3-6].

Gene family clustering analysis were performed using OrthoMCL [35] on all the protein-coding genes of *P. pruinosa* and 10 additional species (*P. euphratica*, *P. trichocarpa*, *Salix suchowensis*, *Ricinus communis*, *Arabidopsis thaliana*, *Carica papaya*, *Fragaria vesca*, *Cucumis sativus*, *Oryza sativa* and *Vitis vinifera*). Of the 35,139 protein-coding genes in *P. pruinosa*, 28,821 (82.02%) could be classified into a total of 17,840 families, with 209 clusters comprising 607 genes being specific to *P. pruinosa* (Additional file 1: Table S13). We identified a total of 6,925 *P. pruinosa*-specific genes, of which 3,596 (51.93%) were supported by gene expression data and/or functional annotation (Additional file 1: Table S14), indicating that there are a large number of species-specific genes even though the genomes of *P. pruinosa* and *P. euphratica* are closely related to each other. Fourfold degenerate sites of 1,237 single-copy gene families were extracted and joined into one 'super gene' for each species in order to construct a phylogenetic tree using RAxML [36] (Additional file 1: Figure S7). The MCMCTree program [37] was then applied to estimate the divergence time based on the phylogenetic relationships, using fossil calibration times obtained from the TimeTree database (http://www.timetree.org/). The divergence time between *P. pruinosa* and *P. euphratica* was estimated to be 2.0 (1.0-3.8) million years ago (Additional file 1: Figure S8). Lastly we applied the CAFÉ (Computational Analysis of gene Family Evolution) [38] program to examine gene family evolution across entire genomes. The results showed that 613 gene families related to 'Small molecule metabolic process', 'ADP binding', 'Glucosyltransferase activity', 'Ion channel complex' and 'Lipid transport' were substantially expanded in *P. pruinosa* compared to other plant species (Additional file 1: table S15 and Figure S9). Expansions in these families may be functionally correlated with the specialized desert habitat of *P. pruinosa* [3-6].

In summary, we present here the sequencing, assembly and annotation of the genome of *P. pruinosa*, and compare it with that of its sister species *P. euphratica*. Although a high level of overall similarity was observed between the two genomes, our evolutionary analyses identified a significant number of genes showing signs of adaptive divergence and numerous species-specific genes. The large number of genetic

variations recovered is unexpected because of the recent divergence of the two species around two million years ago. These variations may have resulted from rapid habitat adaptation and natural selection during speciation of the two species. However, population genomic analyses will be needed in order to examine whether these variations are widely fixed across all populations of each species. In addition, functional tests should be performed to explore the roles that variations play in both morphological and ecological divergence. Finally, the large number of genomic variations observed here between two closely related species suggest that pan-genome analyses of all poplars at both the species and the population level will be necessary in the future.

Acknowledgement

This project was supported by the National Key Research and Development Program of China (2016YFD0600101) and the National Natural Science Foundation of China (31561123001 and 31500502).

Availability of supporting data

The assembly and annotation of the *P. pruinosa* genome are available at the Salinity Tolerant Poplar Database (http://me.lzu.edu.cn/stpd). The sequencing reads from each sequencing library have been deposited at NCBI with the Project ID: PRJNA353148, Sample ID: SAMN06011208. Supplementary figures and tables are provided in Additional file 1.

Competing interests

The authors declare that they have no competing interests.

References

1. Tuskan GA, Difazio S, Jansson S, Bohlmann J, Grigoriev I, Hellsten U, Putnam N, Ralph S, Rombauts S, Salamov A *et al*: **The genome of black cottonwood, *Populus trichocarpa* (Torr. & Gray)**. *Science* 2006, **313**(5793):1596-1604.

2. Ma T, Wang J, Zhou G, Yue Z, Hu Q, Chen Y, Liu B, Qiu Q, Wang Z, Zhang J *et al*: **Genomic insights into salt adaptation in a desert poplar**. *Nature communications* 2013, **4**.
3. Eckenwalder JE: **Systematics and evolution of *Populus***. *Biology of Populus and its Implications for Management and Conservation* 1996, **7**:30.
4. Dickmann DI, Kuzovkina J: **Poplars and willows of the world, with emphasis on silviculturally important species**. *Poplars and Willows: Trees for Society and the Environment* 2014, **22**:8.
5. Zhang J, Xie P, Lascoux M, Meagher TR, Liu J: **Rapidly evolving genes and stress adaptation of two desert poplars, *Populus euphratica* and *P. pruinosa***. *PloS one* 2013, **8**(6):e66370.
6. Zhang J, Feng J, Lu J, Yang Y, Zhang X, Wan D, Liu J: **Transcriptome differences between two sister desert poplar species under salt stress**. *BMC genomics* 2014, **15**(1):1.
7. Song L, Florea L, Langmead B: **Lighter: fast and memory-efficient sequencing error correction without counting**. *Genome biology* 2014, **15**(11):1.
8. Xu H, Luo X, Qian J, Pang X, Song J, Qian G, Chen J, Chen S: **FastUniq: a fast *de novo* duplicates removal tool for paired short reads**. *PloS one* 2012, **7**(12):e52249.
9. Marçais G, Kingsford C: **A fast, lock-free approach for efficient parallel counting of occurrences of k-mers**. *Bioinformatics* 2011, **27**(6):764-770.
10. Kajitani R, Toshimoto K, Noguchi H, Toyoda A, Ogura Y, Okuno M, Yabana M, Harada M, Nagayasu E, Maruyama H *et al*: **Efficient *de novo* assembly of highly heterozygous genomes from whole-genome shotgun short reads**. *Genome research* 2014, **24**(8):1384-1395.
11. Li R, Li Y, Kristiansen K, Wang J: **SOAP: short oligonucleotide alignment program**. *Bioinformatics* 2008, **24**(5):713-714.
12. Parra G, Bradnam K, Korf I: **CEGMA: a pipeline to accurately annotate core genes in eukaryotic genomes**. *Bioinformatics* 2007, **23**(9):1061-1067.
13. Simão FA, Waterhouse RM, Ioannidis P, Kriventseva EV, Zdobnov EM: **BUSCO: assessing genome assembly and annotation completeness with single-copy orthologs**. *Bioinformatics* 2015:btv351.
14. Li H: **Aligning sequence reads, clone sequences and assembly contigs with BWA-MEM**. *arXiv preprint arXiv:13033997* 2013.
15. DePristo MA, Banks E, Poplin R, Garimella KV, Maguire JR, Hartl C, Philippakis AA, Del Angel G, Rivas MA, Hanna M *et al*: **A framework for variation discovery and genotyping using next-generation DNA sequencing data**. *Nature genetics* 2011, **43**(5):491-498.
16. Tarailo - Graovac M, Chen N: **Using RepeatMasker to identify repetitive elements in genomic sequences**. *Current Protocols in Bioinformatics* 2009:4.10. 1-4.10. 14.

17. Jurka J, Kapitonov VV, Pavlicek A, Klonowski P, Kohany O, Walichiewicz J: **Repbase Update, a database of eukaryotic repetitive elements**. *Cytogenetic and genome research* 2005, **110**(1-4):462-467.
18. Benson G: **Tandem repeats finder: a program to analyze DNA sequences**. *Nucleic acids research* 1999, **27**(2):573.
19. Camacho C, Coulouris G, Avagyan V, Ma N, Papadopoulos J, Bealer K, Madden TL: **BLAST+: architecture and applications**. *BMC bioinformatics* 2009, **10**(1):1.
20. Birney E, Clamp M, Durbin R: **GeneWise and genomewise**. *Genome research* 2004, **14**(5):988-995.
21. Stanke M, Keller O, Gunduz I, Hayes A, Waack S, Morgenstern B: **AUGUSTUS: *ab initio* prediction of alternative transcripts**. *Nucleic acids research* 2006, **34**(suppl 2):W435-W439.
22. Salamov AA, Solovyev VV: ***Ab initio* gene finding in *Drosophila* genomic DNA**. *Genome research* 2000, **10**(4):516-522.
23. Grabherr MG, Haas BJ, Yassour M, Levin JZ, Thompson DA, Amit I, Adiconis X, Fan L, Raychowdhury R, Zeng Q *et al*: **Full-length transcriptome assembly from RNA-Seq data without a reference genome**. *Nature biotechnology* 2011, **29**(7):644-652.
24. Li W, Godzik A: **Cd-hit: a fast program for clustering and comparing large sets of protein or nucleotide sequences**. *Bioinformatics* 2006, **22**(13):1658-1659.
25. Haas BJ, Delcher AL, Mount SM, Wortman JR, Smith Jr RK, Hannick LI, Maiti R, Ronning CM, Rusch DB, Town CD *et al*: **Improving the *Arabidopsis* genome annotation using maximal transcript alignment assemblies**. *Nucleic acids research* 2003, **31**(19):5654-5666.
26. Xu Y, Wang X, Yang J, Vaynberg J, Qin J: **PASA–a program for automated protein NMR backbone signal assignment by pattern-filtering approach**. *Journal of biomolecular NMR* 2006, **34**(1):41-56.
27. Haas BJ, Salzberg SL, Zhu W, Pertea M, Allen JE, Orvis J, White O, Buell CR, Wortman JR: **Automated eukaryotic gene structure annotation using EVidenceModeler and the Program to Assemble Spliced Alignments**. *Genome biology* 2008, **9**(1):1.
28. Bairoch A, Apweiler R: **The SWISS-PROT protein sequence database and its supplement TrEMBL in 2000**. *Nucleic acids research* 2000, **28**(1):45-48.
29. Hunter S, Apweiler R, Attwood TK, Bairoch A, Bateman A, Binns D, Bork P, Das U, Daugherty L, Duquenne L *et al*: **InterPro: the integrative protein signature database**. *Nucleic acids research* 2009, **37**(suppl 1):D211-D215.
30. Kanehisa M, Goto S: **KEGG: kyoto encyclopedia of genes and genomes**. *Nucleic acids research* 2000, **28**(1):27-30.
31. Ashburner M, Ball CA, Blake JA, Botstein D, Butler H, Cherry JM, Davis AP, Dolinski K, Dwight SS, Eppig JT *et al*: **Gene Ontology: tool for the unification of biology**. *Nature genetics* 2000, **25**(1):25-29.

32. Conesa A, Götz S: **Blast2GO: A comprehensive suite for functional analysis in plant genomics**. *International journal of plant genomics* 2008, **2008**.
33. Wang Y, Tang H, DeBarry JD, Tan X, Li J, Wang X, Lee T-h, Jin H, Marler B, Guo H *et al*: **MCScanX: a toolkit for detection and evolutionary analysis of gene synteny and collinearity**. *Nucleic acids research* 2012, **40**(7):e49-e49.
34. Qiu Q, Zhang G, Ma T, Qian W, Wang J, Ye Z, Cao C, Hu Q, Kim J, Larkin DM *et al*: **The yak genome and adaptation to life at high altitude**. *Nature genetics* 2012, **44**(8):946-949.
35. Li L, Stoeckert Jr. CJ, Roos DS: **OrthoMCL: Identification of Ortholog Groups for Eukaryotic Genomes**. *Genome Res* 2003, **13**(1):2178–2189.
36. Stamatakis A: **RAxML version 8: a tool for phylogenetic analysis and post-analysis of large phylogenies**. *Bioinformatics* 2014, **30**(9):1312-1313.
37. Yang Z: **PAML 4: phylogenetic analysis by maximum likelihood**. *Molecular biology and evolution* 2007, **24**(8):1586-1591.
38. De Bie T, Cristianini N, Demuth JP, Hahn MW: **CAFE: a computational tool for the study of gene family evolution**. *Bioinformatics* 2006, **22**(10):1269-1271.

Additional file

Additional file 1: Supplementary tables and figures.

Table S1: Summary of qualified reads after the raw reads from the Illumina platform had been filtered using Lighter and FastUniq.

Table S2: Estimation of *P. pruinosa* genome size based on 17-mer statistics.

Table S3: Statistics on the final assembly of the *P. pruinosa* genome

Table S4: Gene region coverage assessed by CEGMA.

Table S5: Summary of BUSCO analysis.

Table S6: Prediction of repetitive elements in the *P. pruinosa* genome.

Table S7: Classification of repetitive elements in the *P. pruinosa* genome.

Table S8: Statistics on *P. pruinosa* transcriptome sequencing and read alignments.

Table S9: Statistics on predicted protein-coding genes in the *P. pruinosa* genome.

Table S10: Functional annotation of predicted genes in *P. pruinosa.*

Table S11: Summary of collinear blocks between *P. pruinosa* and *P. euphratica*.

Table S12: Top 10 GO categories (biological process and molecular function) displaying the highest Ka/Ks ratios between *P. pruinosa* and *P. euphratica*.

Table S13: Summary of gene family clustering.

Table S14. Analysis of *P. pruinosa* species-specific genes.

Table S15: GO enrichment analysis for expanded gene families in the *P. pruinosa* genome.

Figure S1: 17-mer analysis for estimating *P. pruinosa* genome size based on reads from short insert libraries.

Figure S2: GC content distribution for the genome of *P. pruinosa* and related poplar species, established by 500 bp non-overlapping sliding windows.

Figure S3: Sequencing depth distribution for the *P. pruinosa* genome.

Figure S4: Comparison of mRNA length (A), CDS length (B), Exon length (C), Intron length (D), and Exon number per gene (E) in *P. pruinosa* and in related poplar species.

Figure S5: Genome duplication in poplar genomes as revealed by Ks analyses.

Figure S6: Distribution of Ka, Ks, Ka/Ks and protein similarity in 1:1 *P. pruinosa*-*P. euphratica* orthologs within syntenic blocks.

Figure S7: Phylogenetic relationships of *P. pruinosa* and 10 other species.

Figure S8: Estimation of divergence time using phylogenetic analysis.

Figure S9: Dynamic evolution of orthologous gene families.

Click here to access/download
Supplementary Material
PprGenome-V6-supplement.docx
