# Supplementary material for: The draft genome sequence of a desert tree Populus pruinosa
Source: Gigascience. 2017 Aug 8;6(9):1–7. doi: 10.1093/gigascience/gix075 (PMC5603765; doi:10.1093/gigascience/gix075)
Supplement: GIGA-D-16-00147_Revision-1.pdf [file gix075_GIGA-D-16-00147_Revision-1.pdf]

Manuscript

Click here to download Manuscript PprGenome-V10-mauscript.pdf

The draft genome sequence of a desert tree *Populus pruinosa*

Wenlu Yang[1], Kun Wang[1], Jian Zhang[2], Jianchao Ma[2], Jianquan Liu[1,2], Tao Ma[1*]

[1]MOE Key Laboratory for Bio-resources and Eco-environment, College of Life Science, Sichuan University, Chengdu, China

[2]State Key Laboratory of Grassland Agro-Ecosystem, College of Life Science, Lanzhou University, Lanzhou, China

*Correspondence should be addressed to T. M. (matao.yz@gmail.com)

Abstract

Background

Populus pruinosa is a large tree that grows in deserts and shows distinct differences in both morphology and adaptation from those of its sister species, *P. euphratica*. Here we present a draft genome sequence for *P. pruinosa* and examine genomic variations between the two species.

Findings

A total of 60 Gb of clean reads from whole-genome sequencing of a *P. pruinosa* individual were generated using the Illumina HiSeq2000 platform. The assembled genome is 479.3 Mb in length, with an N50 contig size of 14.0 kb and a scaffold size of 698.5 kb. 45.47% of the genome is composed of repetitive elements. We predicted 35,131 protein-coding genes, of which 88.06% were functionally annotated. Gene family clustering revealed 224 unique and 640 expanded gene families in the *P. pruinosa* genome. Further evolutionary analysis identified numerous genes with elevated values for pairwise genetic differentiation between *P. pruinosa* and *P. euphratica*.

Conclusions

We provide the genome sequence and gene annotation for *P. pruinosa*. A large number of genetic variations were recovered by comparison of the genomes between *P. pruinosa* and *P. euphratica*. These variations will provide a valuable resource for studying the genetic bases for the phenotypic and adaptive divergence of the two sister species.

Keywords

Populus pruinosa, Illumina sequencing, Genome assembly, Annotation

Background

Poplars (*Populus* spp.) are widely distributed and cultivated, and they have both economic and ecological importance. Many resequencing based studies have been conducted to identify genetic variations responsible for their phenotypic and adaptive diversity observed in nature [1-4] . However, comparative studies based on *de novo* genome assemblies are still in their infancy, since presently only two reference genomes are available for poplar species, namely *P. trichocarpa* (Torr. & Gray) [5] and *P. euphratica* Oliv. [6]. Further development of genome resources will offer a unique opportunity for comparative genomics and evolutionary studies within this tree genus. *P. pruinosa* Schrenk, the sister species of *P. euphratica* [7], is a large tree distributed in the deserts of western China and adjacent regions [8]. These two species are morphologically well differentiated. The leaves of *P. pruinosa* are ovate or kidney-shaped with thick hairs, whereas *P. euphratica* has glabrous leaves with heteroblastic development. Although both species are well adapted to extreme desert environments, they grow in the distinct desert habitats: *P. pruinosa* is distributed in deserts where there is highly saline underground water close to the surface, while *P. euphratica* occurs in dry deserts in which the water is deep underground and less saline [8-10]. Previous comparisons of the transcriptomes of these two sister species suggest that they may have developed enough genetic divergence to make it possible for them to adapt to these distinct desert habitats [9, 10]. Genomic resources and comparative genomic analysis of these two species would accelerate our understanding of the processes of genomic evolution underlying their phenotypic and adaptive divergence. Here we report a draft genome assembly for *P. pruinosa* and present an initial comparative genomics analysis of *P. pruinosa* and *P. euphratica*. We recovered a large number of genetic variations including high level of heterozygosity, several genes undergone rapid evolution and numerous gene families unique and expanded in *P. pruinosa* genome.

Data description

Samples and Sequencing

High-quality genomic DNA was extracted from the leaf tissues of a single *P. pruinosa* tree (NCBI Taxonomy ID: 492479) collected in Xinjiang, China, using the cetyl trimethylammonium bromide (CTAB) method. Sequencing libraries with different insert sizes were constructed according to the Illumina protocol. Briefly, for paired-end libraries with insert sizes ranging from 158 to 780 bp, DNA was fragmented, end repaired, A-tailed and ligated to Illumina paired-end adapters (Illumina). The ligated fragments were size selected on agarose gel and amplified by ligation-mediated PCR to produce the corresponding libraries. For mate pair libraries (2 to 20 kb), about 20-50 μg genomic DNA was fragmented using nebulization for 2 kb or HydroShear (Covaris) for 5, 10 and 20 kb. Next, the DNA fragments were end-repaired using biotinylated nucleotide analogues and purified using QIAquick PCR Purification Kit (Qiagen). Then the target fragments were selected on agarose gel and circularized by intramolecular ligation. Circular DNA was fragmented (Covaris) and biotinylated fragments were purified with magnetic beads (Invitrogen), end-repaired, A-tailed and ligated to Illumina paired-end adapters, size-selected again and purified with QIAquick Gel Extraction kit (QIAGEN). All of the above libraries were sequenced on an Illumina HiSeq 2000 platform. For the data filtering process, we discarded reads that met either of the following criteria: (1) reads with $\geqslant$ 10% unidentified nucleotides; (2) reads from paired-end libraries having more than 40% bases with Phred quality < 8, and reads from mate pair libraries that contained more than 60% bases with the quality < 8; (3) reads with more than 10 bp aligned to the adapter sequence, allowing < 4 bp mismatch; (4) reads from paired-end libraries that overlapped $\geq$ 10 bp with the corresponding paired end. We also corrected the reads containing sequencing errors and removed the duplicates introduced by PCR amplification in paired reads using Lighter v1.0.7 [11] and FastUniq v1.1 [12], respectively. Finally, ~60 Gb of clean data (Additional file 1: Table S1) were obtained for the *de novo* assembly of the *P. pruinosa* genome.

Clean reads obtained from paired-end libraries were subjected to 17-mer frequency distribution analysis with KmerFreq_AR [13]. Analysis parameters were set at -k 17 -t 10 -q 33, and the final result was plotted as a frequency graph (Additional file 1: Figure S1). Two distinctive peaks observed from the distribution curve demonstrated the high heterozygosity of the *P. pruinosa* genome. To prevent the deviation of *k*-mer based methods on the estimation of genome size, we determined the genome size of *P. pruinosa* with flow cytometry, using *Vigna radiata* as reference standard and propidium iodide as the stain. Our flow cytometry analysis showed that the genome size of *P. pruinosa* was approximately 590 Mb (Additional file 1: Figure S2).

In addition, we extracted RNA from leaf, phloem and xylem tissues of a 2-year-old *P. pruinosa* seedling using CTAB method [14]. Then, RNA-seq libraries were constructed using NEB Next Ultra Directional RNA Library Prep Kit for Illumina (NEB, Ispawich, USA) according to the manufacturer's instructions, and libraries were sequenced using an Illumina HiSeq 2500 platform with a read length of 2×125 bp. Over 38 million paired-end reads were generated for each sample (Additional file 1: Table S2). We next assembled these RNA-seq reads using Trinity v2.1.1 [15] with the default parameters and reduced the redundancy of transcript sequences (>95% similarity) using CD-Hit v4.6.1 [16]. The software TransDecoder v2.1.0 [17] was used to identify candidate coding regions within these transcript sequences. Finally, a total of 111,538 unigenes were obtained for subsequent evaluation of gene space completeness of our genome assembly and transcriptome-based gene prediction.

Genome assembly

The *P. pruinosa* genome was *de novo* assembled by Platanus v1.2.1 [18] with default parameter (-k 32), which is optimized for highly heterozygous diploid genomes. Briefly, the clean reads derived from paired-end libraries were firstly split into *k*-mers to construct *de Bruijn* graphs and merged into distinct contigs based on overlap information. All reads from paired-end and mate pair libraries were then aligned against

the contigs and the paired relationships were used to link contigs into scaffolds. Finally, the intra-scaffold gaps were closed by local assembly implemented in GapCloser v1.12 [19] using the paired-end reads for which one end uniquely mapped to a contig but the other end was located within a gap. After discarding the scaffolds smaller than 200 bp, we yielded a draft assembly with a total length of 479.3 Mb (Table 1), which covers 85% of the predicted genome size of *P. pruinosa*. The contig and scaffold N50 sizes were 14.0 kb and 698.5 kb respectively, while the unclosed gap regions represent 6.08% of the assembly (Additional file 1: Table S3). The distribution of the average GC content of the *P. pruinosa* genome (mean: 31.8%) is similar to that for the *P. euphratica* genome [6] (32.1%) and the *P. trichocarpa* genome [5] (33.6%) (Additional file 1: Figure S3).

To evaluate the completeness of this assembly, we first examined the coverage of highly conserved genes using BUSCO [20]. The result showed that 922 out of the 956 conserved genes (96.44%) could be found in our assembly, of which 699 were single and 223 were duplicated, and only 10 (1.05%) genes had fragmented matches (Additional file 1: Table S4). These coverage values were comparable to estimates for the *P. euphratica* and *P. trichocarpa* genomes. Furthermore, the 111,538 *P. pruinosa* unigenes obtained in this study and the protein-coding genes predicted in the *P. euphratica* and *P. trichocarpa* genomes [5, 6] were aligned to our genome assembly using the BLAT algorithm with default parameters. Statistics analysis were done at different levels of percentage of sequence homology and percentage of coverage. The results showed that our assembly covered approximately 90% of the *P. pruinosa* unigenes, 99% and 98% of the protein-coding genes in *P. euphratica* and *P. trichocarpa* respectively (Additional file 1: Table S5). Finally, we applied the FRC v1.3.0 (Feature-Response Curves) method [21] to evaluate the trade-off between the contiguity and correctness of our assembly. This method is based on a prediction of assembly correctness by identifying on each *de novo* assembled scaffold, ‘features’ representing potential errors or complications during the assembly process. Comparison of the three poplar species genomes indicated that our *P. pruinosa* genome assembly generated a

better FRCurve than the other assemblies (Additional file 1: Figure S4). In summary, all of these statistics revealed that our draft genome sequence has high contiguity, accuracy, and more important, high degree of gene space completeness for effective gene detection.

We mapped the clean reads from the paired-end libraries to the *P. pruinosa* genome using the Burrows-Wheeler Aligner (BWA v0.7.12-r1044) [22] and found that the sequencing depth for 95.3% of the assembly was more than 20-fold (Additional file 1: Figure S5), ensuring a high level of accuracy at the nucleotide level. We also performed variant calling using the Genome Analysis Toolkit (GATK v3.5) [23]. A total of 3.11 million heterozygous single nucleotide variants (SNVs) were obtained after strict quality control and filtering, which revealed that the heterozygosity level of the *P. pruinosa* genome was approximately 0.80%.

Repeat annotation

Repetitive sequences and transposable elements (TEs) in the *P. pruinosa* genome were identified using a combination of *de novo* and homology-based approaches at both the DNA and the protein level. Initially, we built a *de novo* repeat library for *P. pruinosa* using RepeatModeler v1.0.8 [24] with default parameters. For identification and classification of transposable elements at the DNA level, RepeatMasker [24] was applied to map our assembly against both the databases that we had built and the known Repbase [25] transposable element (TE) library. Next we executed RepeatProteinMask [24] using a WU-BLASTX search against the TE protein database to further identify repeats at the protein level. In addition, we annotated tandem repeats using the software Tandem Repeat Finder (TRF v4.07b) [26]. In total, the combined non-redundant results showed that approximately 45% of the *P. pruinosa* genome assembly is composed of repetitive elements (Additional file 1: Table S6), a value similar to that for the *P. euphratica* genome (44%). Long terminal repeats (LTRs) were the most abundant repeat class, accounting for 67.03% of repetitive sequences representing 29.82% of the

genome (Additional file 1: Table S7).

Gene annotation

We conducted the gene annotation in the *P. pruinosa* genome by combining homology-based, *de novo* and transcriptome-based methods. For homology-based prediction, protein sequences from six sequenced plants (*P. euphratica* [6], *P. trichocarpa* [5], *Ricinus communis* [27], *Arabidopsis thaliana* [28], *Carica papaya* [29] and *Eucalyptus grandis* [30]) were aligned to the *P. pruinosa* genome using TBLASTN v2.2.26 [31]. The homologous genome sequences were then aligned against the matching proteins using GeneWise v2.4.1 [32] to obtain accurate spliced alignments. For *de novo* prediction, we performed Augustus v3.2.1 [33] and GenScan [34] analysis on the repeat-masked genome with parameters trained from *P. pruinosa* and *A. thaliana*. The resultant data sets were filtered with the removal of partial sequences and genes with coding length less than 100 bp. For transcriptome-based approach, the 111,538 *P. pruinosa* transcripts obtained above were aligned to the *P. pruinosa* genome and further assembled using the Program to Assemble Spliced Alignments (PASA v2.0.2) [35] to detect likely protein coding regions. Finally, we combined the gene annotation results from all homology-based, *de novo* and transcriptome-based predictions using EVM v1.1.1 [36] to produce a consensus protein-coding gene set.

In sum, the *P. pruinosa* genome contains 35,131 protein-coding genes with an average CDS length of 1,224 bp (Additional file 1: Table S8). The length distributions of transcripts, coding sequences, exons and introns were similar in *P. euphratica* and in *P. trichocarpa* (Additional file 1: Figure S6). Functional annotation was performed based on comparisons with the SwissProt, TrEMBL [37], InterPro [38] and KEGG [39] protein databases. Gene Ontology (GO) [40] IDs for each gene were assigned by the Blast2GO pipeline [41] based on NCBI databases. Overall, 75.43% of the protein-coding genes had conserved protein domains and 63.64% could be classified by GO terms (Additional file 1: Table S9).

Evolutionary analysis

Blocks syntenic between *P. pruinosa* and *P. euphratica* were determined by the software MCScanX [42], at least five genes were required to call synteny. The blocks identified occupy the majority of the genome assemblies of *P. pruinosa* (290 Mb, 66% of the assembly; 29,015 genes, 83% of the predicted gene models) and *P. euphratica* (293 Mb, 59%; 27,804 genes, 81%) (Additional file 1: Table S10), suggesting that there is extensive macrosynteny between these two species. This overall high level of synteny was also confirmed by whole-genome alignment using the program 'LAST' [43] (Fig. 1). A total of 15,695 high-confidence 1:1 orthologous genes were identified in these syntenic blocks. We estimated and plotted the nucleotide synonymous substitution (Ks) rates for these orthologous pairs, and a peak at around 0.016 was observed (Additional file 1: Figure S7), while the divergence between duplicated genes in *P. pruinosa* and *P. euphratica* peaked around 0.272 and 0.257, respectively, indicating that the two species had shared common whole genome duplication (WGD) events before they diverged from a common ancestor. Adaptive divergence at the molecular level may be reflected in an increased rate of nonsynonymous changes within genes involved in adaptation [44]. We found that the mean similarity between *P. euphratica* and *P. pruinosa* orthologous genes at the protein level is close to 97.22% (Additional file 1: Figure S8). Average synonymous (Ks) and nonsynonymous (Ka) gene divergence values were 0.04 and 0.017 respectively. The genes that showed elevated pairwise genetic differentiation were enriched mainly in 'metal ion transport', 'regulation of gene expression', 'response to stimulus', 'antiporter activity', 'heat shock protein binding' and 'oxidoreductase activity' terms (Additional file 1: Table S11), indicating that these functions had undergone rapid evolution (caused by adaptive divergence and/or relaxed selection) between *P. pruinosa* and *P. euphratica*.

Gene family clustering analysis were performed using OrthoMCL v3.1 [45] on all the protein-coding genes of *P. pruinosa* and 10 additional species (*P. euphratica*, *P. trichocarpa*, *Salix suchowensis*, *Ricinus communis*, *Arabidopsis thaliana*, *Carica*

papaya, *Fragaria vesca*, *Cucumis sativus*, *Eucalyptus Grandis* and *Vitis vinifera*). Of the 35,131 protein-coding genes in *P. pruinosa*, 28,773 (81.9%) could be classified into a total of 17,592 families, with 224 clusters comprising 662 genes being specific to *P. pruinosa* (Additional file 1: Table S12). We identified a total of 7,020 *P. pruinosa*-specific genes, of which 3,639 (51.8%) were supported by gene expression data (RPKM > 0.5) and/or functional annotation (Additional file 1: Table S13), indicating that there are a large number of species-specific genes even though the genomes of *P. pruinosa* and *P. euphratica* are closely related to each other. Further analysis revealed that these *P. pruinosa*-specific genes were primarily enriched in 'transcription factor activity', 'transporter activity', 'response to salt stress' and 'oxidoreductase activity' (Additional file 1: Table S14).

In addition, we identified a total of 1,354 single-copy gene families across the 11 plant genomes. Alignments were generated for each family with MUSCLE v3.8.31 [46] and low quality regions of the alignments were identified and trimmed with Gblocks v0.91b [47, 48] using default parameters. The individual trimmed protein-coding alignments were concatenated into one 'super gene' for each species in order to construct a phylogenetic tree using RAxML v8.2.8 [49] (Additional file 1: Figure S9). Then MCMCTree v4.9 [50] was applied to estimate the divergence time based on the phylogenetic relationships, using fossil calibration times for divergence between *A. thaliana* and *C. papaya* (54-90 million years ago, Mya), *A. thaliana* and *R. communis* (95-109 Mya), *V. vinifera* and *A. thaliana* (106-119 Mya), which were obtained from the TimeTree database (http://www.timetree.org/). The divergence time between *P. pruinosa* and *P. euphratica* was estimated to be 3.0 (1.6-5.0) Mya (Additional file 1: Figure S10). Lastly we applied the CAFÉ (Computational Analysis of gene Family Evolution, v3.1) [51] program to examine gene family evolution across entire genomes. The results showed that 640 gene families related to 'Glucosyltransferase activity', 'ADP binding', 'Cation channel activity', 'Cell differentiation' and 'Oxidoreductase activity' were substantially expanded in *P. pruinosa* compared to other plant species (Additional file 1: Table S15 and Figure S11).

In summary, we present here the sequencing, assembly and annotation of the genome of *P. pruinosa*, and compare it with that of its sister species *P. euphratica*. Although a high level of overall similarity was observed between the two genomes, our evolutionary analyses identified a large number of genes showing signs of rapid divergence and numerous species-specific genes, which may have resulted from rapid habitat adaptation and natural selection during speciation of the two species. However, population genomic analyses will be needed in order to examine whether these variations are widely fixed across all populations of each species. In addition, functional tests should be performed to explore the roles that variations play in both morphological and ecological divergence.

Acknowledgement

This project was supported by the National Key Research and Development Program of China (2016YFD0600101), the National Key Project for Basic Research (2012CB114504), the National Natural Science Foundation of China (31561123001 and 31500502) and the Fundamental Research Funds for the Central Universities.

Availability of supporting data

The sequencing reads from each sequencing library have been deposited at NCBI with the Project ID: PRJNA353148, Sample ID: SAMN06011208. The assembly and annotation of the *P. pruinosa* genome, the assembly pipeline and commands used in this work are available in the *GigaScience* database, GigaDB. All supplementary figures and tables are provided in Additional file 1.

Competing interests

The authors declare that they have no competing interests.

References

1. Evans LM, Slavov GT, Rodgers-Melnick E, Martin J, Ranjan P, Muchero W, Brunner AM, Schackwitz W, Gunter L, Chen JG *et al*: **Population genomics of *Populus trichocarpa* identifies signatures of selection and adaptive trait associations**. *Nature genetics* 2014, **46**(10):1089-1096.
2. Wang J, Street NR, Scofield DG, Ingvarsson PK: **Variation in linked selection and recombination drive genomic divergence during allopatric speciation of european and american aspens**. *Molecular biology and evolution* 2016, **33**(7):1754-1767.
3. Pinosio S, Giacomello S, Faivre-Rampant P, Taylor G, Jorge V, Le Paslier MC, Zaina G, Bastien C, Cattonaro F, Marroni F *et al*: **Characterization of the poplar pan-genome by genome-wide identification of structural variation**. *Molecular biology and evolution* 2016, **33**(10):2706-2719.
4. Christe C, Stolting KN, Paris M, Fraïsse C, Bierne N, Lexer C: **Adaptive evolution and segregating load contribute to the genomic landscape of divergence in two tree species connected by episodic gene flow**. *Molecular Ecology* 2017, **26**(1):59-76.
5. Tuskan GA, Difazio S, Jansson S, Bohlmann J, Grigoriev I, Hellsten U, Putnam N, Ralph S, Rombauts S, Salamov A *et al*: **The genome of black cottonwood, *Populus trichocarpa* (Torr. & Gray)**. *Science* 2006, **313**(5793):1596-1604.
6. Ma T, Wang J, Zhou G, Yue Z, Hu Q, Chen Y, Liu B, Qiu Q, Wang Z, Zhang J: **Genomic insights into salt adaptation in a desert poplar**. *Nature communications* 2013, **4**.
7. Eckenwalder JE: **Systematics and evolution of *Populus***. *Biology of Populus and its Implications for Management and Conservation* 1996, **7**:30.
8. Dickmann DI, Kuzovkina J: **Poplars and willows of the world, with emphasis on silviculturally important species**. *Poplars and Willows: Trees for Society and the Environment* 2014, **22**:8.
9. Zhang J, Xie P, Lascoux M, Meagher TR, Liu J: **Rapidly evolving genes and stress adaptation of two desert poplars, *Populus euphratica* and *P. pruinosa***. *PloS one* 2013, **8**(6):e66370.
10. Zhang J, Feng J, Lu J, Yang Y, Zhang X, Wan D, Liu J: **Transcriptome differences between two sister desert poplar species under salt stress**. *BMC genomics* 2014, **15**(1):1.
11. Song L, Florea L, Langmead B: **Lighter: fast and memory-efficient sequencing error correction without counting**. *Genome biology* 2014, **15**(11):1.
12. Xu H, Luo X, Qian J, Pang X, Song J, Qian G, Chen J, Chen S: **FastUniq: a fast *de novo* duplicates removal tool for paired short reads**. *PloS one* 2012, **7**(12):e52249.
13. Marçais G, Kingsford C: **A fast, lock-free approach for efficient parallel counting of occurrences of k-mers**. *Bioinformatics* 2011, **27**(6):764-770.
14. Chang S, Puryear J, Cairney J: **A simple and efficient method for isolating RNA from pine trees**. *Plant molecular biology reporter* 1993, **11**(2):113-116.
15. Grabherr MG, Haas BJ, Yassour M, Levin JZ, Thompson DA, Amit I, Adiconis X, Fan L, Raychowdhury R, Zeng Q: **Full-length transcriptome assembly from RNA-Seq data without a reference genome**. *Nature biotechnology* 2011, **29**(7):644-652.
16. Li W, Godzik A: **Cd-hit: a fast program for clustering and comparing large sets of protein or nucleotide sequences**. *Bioinformatics* 2006, **22**(13):1658-1659.

17. Haas BJ, Delcher AL, Mount SM, Wortman JR, Smith Jr RK, Hannick LI, Maiti R, Ronning CM, Rusch DB, Town CD: **Improving the *Arabidopsis* genome annotation using maximal transcript alignment assemblies**. *Nucleic acids research* 2003, **31**(19):5654-5666.

18. Kajitani R, Toshimoto K, Noguchi H, Toyoda A, Ogura Y, Okuno M, Yabana M, Harada M, Nagayasu E, Maruyama H: **Efficient *de novo* assembly of highly heterozygous genomes from whole-genome shotgun short reads**. *Genome research* 2014, **24**(8):1384-1395.

19. Li R, Li Y, Kristiansen K, Wang J: **SOAP: short oligonucleotide alignment program**. *Bioinformatics* 2008, **24**(5):713-714.

20. Simão FA, Waterhouse RM, Ioannidis P, Kriventseva EV, Zdobnov EM: **BUSCO: assessing genome assembly and annotation completeness with single-copy orthologs**. *Bioinformatics* 2015:btv351.

21. Vezzi F, Narzisi G, Mishra B: **Reevaluating assembly evaluations with Feature Response Curves: GAGE and Assemblathons**. *PLoS one* 2012, **7**(12):e52210.

22. Li H: **Aligning sequence reads, clone sequences and assembly contigs with BWA-MEM**. *arXiv preprint arXiv:13033997* 2013.

23. DePristo MA, Banks E, Poplin R, Garimella KV, Maguire JR, Hartl C, Philippakis AA, Del Angel G, Rivas MA, Hanna M: **A framework for variation discovery and genotyping using next-generation DNA sequencing data**. *Nature genetics* 2011, **43**(5):491-498.

24. Tarailo-Graovac M, Chen N: **Using RepeatMasker to identify repetitive elements in genomic sequences**. *Current Protocols in Bioinformatics* 2009:4-10.

25. Jurka J, Kapitonov VV, Pavlicek A, Klonowski P, Kohany O, Walichiewicz J: **Repbase Update, a database of eukaryotic repetitive elements**. *Cytogenetic and genome research* 2005, **110**(1-4):462-467.

26. Benson G: **Tandem repeats finder: a program to analyze DNA sequences**. *Nucleic acids research* 1999, **27**(2):573.

27. Chan AP, Crabtree J, Zhao Q, Lorenzi H, Orvis J, Puiu D, Melake-Berhan A, Jones KM, Redman J, Chen G *et al*: **Draft genome sequence of the oilseed species *Ricinus communis***. *Nature biotechnology* 2010, **28**(9):951-956.

28. Arabidopsis Genome Initiative. **Analysis of the genome sequence of the flowering plant *Arabidopsis thaliana***. *Nature* 2000, **408**(6814):796-815.

29. Ming R, Hou S, Feng Y, Yu Q, Dionne-Laporte A, Saw JH, Senin P, Wang W, Ly BV, Lewis KLT *et al*: **The draft genome of the transgenic tropical fruit tree papaya (*Carica papaya* Linnaeus)**. *Nature* 2008, **452**(7190):991-996.

30. Myburg AA, Grattapaglia D, Tuskan GA, Hellsten U, Hayes RD, Grimwood J, Jenkins J, Lindquist E, Tice H, Bauer D *et al*: **The genome of *Eucalyptus grandis***. *Nature* 2014, **510**(7505):356-362.

31. Camacho C, Coulouris G, Avagyan V, Ma N, Papadopoulos J, Bealer K, Madden TL: **BLAST+: architecture and applications**. *BMC bioinformatics* 2009, **10**(1):1.

32. Birney E, Clamp M, Durbin R: **GeneWise and genomewise**. *Genome research* 2004, **14**(5):988-995.

33. Stanke M, Keller O, Gunduz I, Hayes A, Waack S, Morgenstern B: **AUGUSTUS: *ab initio* prediction of alternative transcripts**. *Nucleic acids research* 2006, **34**:W435-W439.

34. Salamov AA, Solovyev VV: ***Ab initio* gene finding in *Drosophila* genomic DNA**. *Genome research* 2000, **10**(4):516-522.

35. Xu Y, Wang X, Yang J, Vaynberg J, Qin J: **PASA–a program for automated protein NMR backbone signal assignment by pattern-filtering approach**. *Journal of biomolecular NMR* 2006, **34**(1):41-56.

36. Haas BJ, Salzberg SL, Zhu W, Pertea M, Allen JE, Orvis J, White O, Buell CR, Wortman JR: **Automated eukaryotic gene structure annotation using EVidenceModeler and the Program to Assemble Spliced Alignments**. *Genome biology* 2008, **9**(1):1.

37. Bairoch A, Apweiler R: **The SWISS-PROT protein sequence database and its supplement TrEMBL in 2000**. *Nucleic acids research* 2000, **28**(1):45-48.

38. Hunter S, Apweiler R, Attwood TK, Bairoch A, Bateman A, Binns D, Bork P, Das U, Daugherty L, Duquenne L: **InterPro: the integrative protein signature database**. *Nucleic acids research* 2009, **37**:D211-D215.

39. Kanehisa M, Goto S: **KEGG: kyoto encyclopedia of genes and genomes**. *Nucleic acids research* 2000, **28**(1):27-30.

40. Ashburner M, Ball CA, Blake JA, Botstein D, Butler H, Cherry JM, Davis AP, Dolinski K, Dwight SS, Eppig JT: **Gene Ontology: tool for the unification of biology**. *Nature genetics* 2000, **25**(1):25-29.

41. Conesa A, Götz S: **Blast2GO: A comprehensive suite for functional analysis in plant genomics**. *International journal of plant genomics* 2008, **2008**.

42. Wang Y, Tang H, DeBarry JD, Tan X, Li J, Wang X, Lee T-h, Jin H, Marler B, Guo H: **MCScanX: a toolkit for detection and evolutionary analysis of gene synteny and collinearity**. *Nucleic acids research* 2012, **40**(7):e49-e49.

43. Kiełbasa SM, Wan R, Sato K, Horton P, Frith MC: **Adaptive seeds tame genomic sequence comparison**. *Genome research* 2011, **21**(3):487-493.

44. Qiu Q, Zhang G, Ma T, Qian W, Wang J, Ye Z, Cao C, Hu Q, Kim J, Larkin DM: **The yak genome and adaptation to life at high altitude**. *Nature genetics* 2012, **44**(8):946-949.

45. Li L, Stoeckert Jr. CJ, Roos DS: **OrthoMCL: Identification of Ortholog Groups for Eukaryotic Genomes**. *Genome Res* 2003, **13**(1):2178–2189.

46. Edgar RC: **MUSCLE: a multiple sequence alignment method with reduced time and space complexity**. *BMC Bioinformatics* 2004, **5**:113-113.

47. Castresana J: **Selection of Conserved Blocks from Multiple Alignments for Their Use in Phylogenetic Analysis**. *Molecular Biology and Evolution* 2000, **17**(4):540-552.

48. Talavera G, Castresana J: **Improvement of Phylogenies after Removing Divergent and Ambiguously Aligned Blocks from Protein Sequence Alignments**. *Systematic Biology* 2007, **56**(4):564-577.

49. Stamatakis A: **RAxML version 8: a tool for phylogenetic analysis and post-analysis of large phylogenies**. *Bioinformatics* 2014, **30**(9):1312-1313.

50. Yang Z: **PAML 4: phylogenetic analysis by maximum likelihood**. *Molecular biology and evolution* 2007, **24**(8):1586-1591.

51. De Bie T, Cristianini N, Demuth JP, Hahn MW: **CAFE: a computational tool for the study of gene family evolution**. *Bioinformatics* 2006, **22**(10):1269-1271.

Additional file

Additional file 1: Supplementary tables and figures.

Table S1: Summary of clean reads after the raw reads from the Illumina platform had been filtered using Lighter and FastUniq.

Table S2: Statistics for *P. pruinosa* RNA-seq data.

Table S3: Statistics for the final assembly of the *P. pruinosa* genome.

Table S4: Summary of BUSCO analysis.

Table S5. Evaluation of gene space completeness for the *P. pruinosa* genome.

Table S6: Prediction of repetitive elements in the *P. pruinosa* genome.

Table S7: Classification of repetitive elements in the *P. pruinosa* genome.

Table S8: Statistics of predicted protein-coding genes in the *P. pruinosa* genome.

Table S9: Functional annotation of predicted genes for *P. pruinosa.*

Table S10: Summary of syntenic blocks between *P. pruinosa* and *P. euphratica* identified using MCScanX.

Table S11: Top 10 GO categories (biological process and molecular function) displaying the highest Ka/Ks ratios between *P. pruinosa* and *P. euphratica*.

Table S12: Summary of gene family clustering.

Table S13. Analysis of *P. pruinosa* species-specific genes.

Table S14: GO enrichment analysis of species-specific genes in the *P. pruinosa* genome.

Table S15: GO enrichment analysis of expanded gene families in the *P. pruinosa* genome.

Figure S1: 17-mer analysis for *P. pruinosa* genome based on clean reads from paired-end libraries.

Figure S2: Flow cytometry estimate of the *P. pruinosa* genome size compared to reference standard of *Vigna radiate* (543Mb).

Figure S3: GC content distribution for the genomes of *P. pruinosa* and related poplar species, established by 500 bp non-overlapping sliding windows.

Figure S4: FRCurve of four genome assemblies.

Figure S5: Sequencing depth distribution for the *P. pruinosa* genome.

Figure S6: Comparison of mRNA length (A), CDS length (B), Exon length (C), Intron length (D), and Exon number per gene (E) in *P. pruinosa* and in related poplar species.

Figure S7: Genome duplication in *Populus* genomes as revealed by Ks analyses.

Figure S8: Distribution of Ka, Ks, Ka/Ks and protein similarity in 1:1 *P. pruinosa*-*P. euphratica* orthologs within syntenic blocks.

Figure S9: Phylogenetic relationships of *P. pruinosa* and 10 other plant species.

Figure S10: Estimation of divergence time between *P. pruinosa* and *P. euphratica* using phylogenetic analysis.

Figure S11: Dynamic evolution of orthologous gene families.

Table Click here to download Table PprGenome-V10-table1.pdf

Table 1. Summary of genome assembly and annotation of *P. pruinosa*.

**Genome assembly**		
**Estimate of genome size**		590 Mb
**GC content**		31.80%
**Contigs**		
	N50 size	14,011 bp
	Longest	197,623 bp
	Total number	170,219
	Total size	450,157,195 bp
**Scaffolds**		
	N50 size	698,525 bp
	Longest	10,688,665 bp
	Total number	78,960
	Total length	479,307,600 bp
**Genome annotation**		
**Transposable elements**		
	LTR	142,923,156 bp (29.82%)
	LINE	4,956,260 bp (1.03%)
	DNA	20,990,612 bp (4.38%)
	Total	213,236,753 bp (45.47%)
**Protein coding genes**		
	Total number	35,131
	Mean transcript length	3703.4 bp
	Mean coding sequence length	1224.38 bp
	Mean exon length	226.27 bp
	Mean intron length	561.98 bp
**Functional annotation**		
	GO	22,361 (63.64%)
	KEGG	11,746 (33.43%)
	Total	30,938 (88.06%)

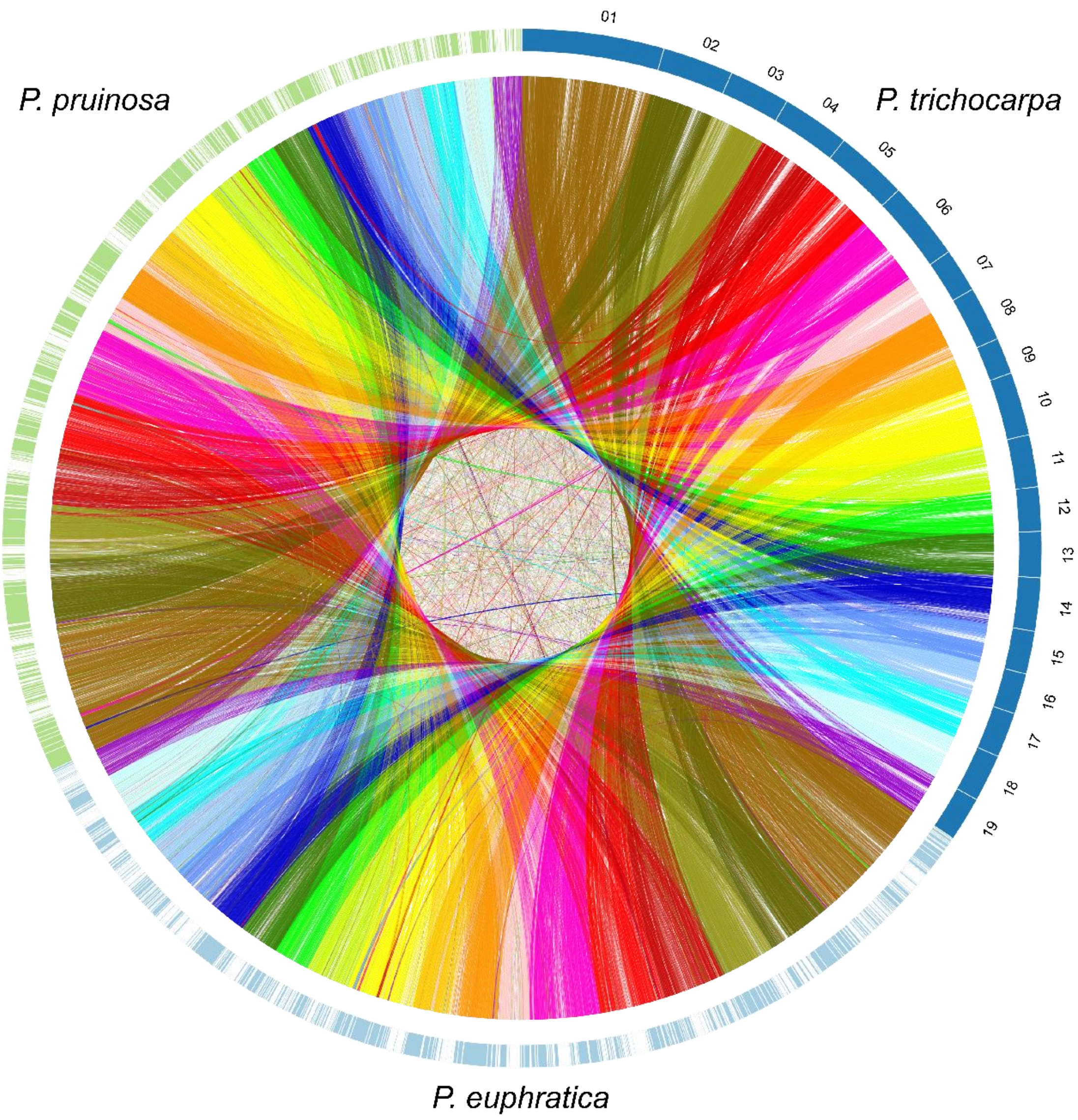

Figure 1. Synteny relationship of *P. pruinosa*, *P. euphratica* and *P. trichocarpa*.

Click here to access/download
Supplementary Material
PprGenome-V10-supplement.pdf

Personal Cover
Click here to download Personal Cover PprGenome-V10-coverLetter.pdf

Dear Editor,

Please find the revised manuscript entitled 'The draft genome sequence of a desert tree *Populus pruinosa*'. We thank you and the three reviewers for the effort and time taken to review our manuscript and for the very useful comments provided.

According to the suggestions of the first reviewer, we have added the genome of *Eucalyptus grandis* for the gene prediction of *P. pruinosa* genome, and re-done almost all the subsequent analysis in our revised manuscript. The detailed information for sequencing library construction and the criterion for low quality read filtering were provided. We also determined the genome size of *P. pruinosa* using flow cytometry and assessed the gene space completeness of our assembly more comprehensively as suggested by the second reviewer. We further uploaded all the data and the analysis pipeline described in this manuscript to *GigaScience* database using the following account:

username = user15
password = MaTaoYZ
ftp://user15@climb.genomics.cn

Having revised the manuscript thoroughly point to point according to the suggestions of the three reviewers, we hope that our manuscript is now more suitable for publication in *GigaScience*. Please do not hesitate to contact us if you require additional information in the context of this submission.

Best wishes

Sincerely yours,

Tao Ma
