# Supplementary material for: The draft genome sequence of a desert tree Populus pruinosa
Source: Gigascience. 2017 Aug 8;6(9):1–7. doi: 10.1093/gigascience/gix075 (PMC5603765; doi:10.1093/gigascience/gix075)
Supplement: Additional Tables [file gix075_Supp.docx]

**Additional tables**

**Table S1: Summary of clean reads after the raw reads from the Illumina platform had been filtered using Lighter** **and FastUniq.**

| **Insert size** | **Types** | **Reads number** | **Reads length (bp)** | **Total length (bp)** | **Depth^1^ (X)** | **Accession number** |
| --- | --- | --- | --- | --- | --- | --- |
| 158 bp | Paired-end | 294,559,178 | 99.86 | 29,414,973,475 | 49.86 | SRR5020682 |
| 483 bp | Paired-end | 130,205,490 | 99.78 | 12,991,870,484 | 22.02 | SRR5020679 |
| 780 bp | Paired-end | 37,477,518 | 100 | 3,747,751,800 | 6.35 | SRR5020683 |
| 2 k | Mate pair | 165,011,632 | 49 | 8,085,569,968 | 13.70 | SRR5020678 |
| 5 k | Mate pair | 65,179,594 | 49 | 3,193,800,106 | 5.41 | SRR5020681 |
| 10 k | Mate pair | 46,125,956 | 49 | 2,260,171,844 | 3.83 | SRR5020680 |
| 20 k | Mate pair | 12,770,036 | 49 | 625,731,764 | 1.06 | SRR5020677 |
| Total | - | 751,329,404 | - | 60,319,869,441 | 102.24 |  |

^1^Depth was calculated under the assumption of a genome size of 590 Mb.

**Table S2: Statistics for *P. pruinosa* RNA-seq data.**

| **Tissue** | **No. cleaned reads** | **No. mapped reads** | **Percentage (%)** | **Accession number** |
| --- | --- | --- | --- | --- |
| leaf | 41,995,923 | 35,444,559 | 84.4 | SRR5055908 |
| phloem | 38,432,556 | 33,705,351 | 87.7 | SRR5055910 |
| xylem | 44,101,662 | 37,574,616 | 85.2 | SRR5055923 |

**Table S3: Statistics for the final assembly of the *P. pruinosa* genome.**

|  | **Contigs** | | **Scaffolds** | |
| --- | --- | --- | --- | --- |
|  | **Size (bp)** | **Number** | **Size (bp)** | **Number** |
| N50^1^ | 14,011 | 8,941 | 698,525 | 135 |
| N60 | 10,679 | 12,622 | 374,511 | 231 |
| N70 | 7,533 | 17,629 | 183,610 | 413 |
| N80 | 4,484 | 25,285 | 62,995 | 849 |
| N90 | 1,429 | 42,256 | 3,918 | 4,093 |
| Longest | 197,623 |  | 10,688,665 |  |
| Gap ratio | 0 (0%) |  | 29,150,405 (6.08%) |  |
| Number > 2 kb |  | 36,911 |  | 6,529 |
| Total | 450,157,195 | 170,219 | 479,307,600 | 78,960 |

^1^N50 refers to the size above which 50% of the total length of the sequence assembly can be found. N60, N70, N80 and N90 are similarly defined.

**Table S4: Summary of BUSCO analysis.**

| **Species** | **Total number of BUSCO groups searched** | **Complete single-copy BUSCOs** | **Complete duplicated BUSCOs** | **Fragmented BUSCOs** | **Missing BUSCOs** |
| --- | --- | --- | --- | --- | --- |
| *P. pruinosa* | 956 | 699 | 223 | 10 | 24 |
| *P. euphratica* | 956 | 652 | 271 | 13 | 20 |
| *P. trichocarpa* | 956 | 682 | 239 | 15 | 20 |

**Table S5. Evaluation of gene space completeness for the *P. pruinosa* genome.**

| Length of unigene | Total number | Total  Length  (bp) | Covered by assemble  (%) |  | With >90% sequence in one scaffold | |  | With >50% sequence in one scaffold | |
| --- | --- | --- | --- | --- | --- | --- | --- | --- | --- |
|  |  |  |  |  | Number | Percent (%) |  | Number | Percent (%) |
| ***P. pruinosa* unigenes assembled from Illumina RNA-seq:** | | | | | | | | | |
| >0 | 111,538 | 97,504,260 | 90.28 |  | 94,173 | 84.43 |  | 95,557 | 85.67 |
| >200 | 111,538 | 97,504,260 | 90.28 |  | 94,173 | 84.43 |  | 95,557 | 85.67 |
| >500 | 70,315 | 81,821,049 | 92.69 |  | 62,717 | 89.19 |  | 63,525 | 90.34 |
| >1000 | 32,556 | 54,975,906 | 95.19 |  | 30,243 | 92.90 |  | 30,652 | 94.15 |
| **Protein-coding sequence predicted in the *P. trichocarpa* genome:** | | | | | | | | | |
| >0 | 41,335 | 47,864,232 | 98.49 |  | 38,043 | 92.04 |  | 40,357 | 97.63 |
| >200 | 40,602 | 47,739,978 | 98.52 |  | 37,481 | 92.31 |  | 39,679 | 97.73 |
| >500 | 30,816 | 44,479,005 | 98.93 |  | 29,501 | 95.73 |  | 30,452 | 98.82 |
| >1000 | 19,636 | 36,211,029 | 99.28 |  | 19,159 | 97.57 |  | 19,526 | 99.44 |
| **Protein-coding sequence predicted in the *P. euphratica* genome:** | | | | | | | | | |
| >0 | 34,279 | 43,615,029 | 98.90 |  | 33,205 | 96.87 |  | 33,895 | 98.88 |
| >200 | 33,637 | 43,501,779 | 98.90 |  | 32,616 | 96.96 |  | 33,267 | 98.90 |
| >500 | 27,432 | 41,317,878 | 98.95 |  | 26,719 | 97.40 |  | 27,155 | 98.99 |
| >1000 | 18,083 | 34,347,219 | 99.03 |  | 17,684 | 97.79 |  | 17,927 | 99.14 |

**Table S6: Prediction of repetitive elements in the** ***P. pruinosa* genome.**

| **Type** | **Repeat Size (bp)** | **% of genome** |
| --- | --- | --- |
| **Tandem Repeat Finder** | 14,904,236 | 3.11 |
| **RepeatMasker** | 155,496,679 | 32.44 |
| **RepeatProteinMask** | 54,001,706 | 11.27 |
| **RepeatModeler** | 196,787,079 | 41.06 |
| **Total^1^** | 217,919,180 | 45.47 |

^1^Total repeat regions were identified combining all the repeats identified. As there are some overlaps between different methods, the total region is shorter than the sum of repeats identified by all methods.

**Table S7: Classification of repetitive elements in the *P. pruinosa* genome.**

| **Type** | **Combined^1^ TE** | | |  | **RepeatMasker** | | |  | ***De novo*** | | |  | **RepeatProteinMask** | | |
| --- | --- | --- | --- | --- | --- | --- | --- | --- | --- | --- | --- | --- | --- | --- | --- |
|  | **Repeat size (bp)** | **% of Repeats** | **% of genome** |  | **Repeat size (bp)** | **% of Repeats** | **% of genome** |  | **Repeat size (bp)** | **% of Repeats** | **% of genome** |  | **Repeat size (bp)** | **% of Repeats** | **% of genome** |
| **DNA** | 20,990,612 | 9.84 | 4.38 |  | 13,816,978 | 8.89 | 2.88 |  | 17,361,228 | 8.82 | 3.62 |  | 4,575,325 | 8.47 | 0.95 |
| **LINE** | 4,956,260 | 2.32 | 1.03 |  | 3,171,642 | 2.04 | 0.66 |  | 2,629,803 | 1.34 | 0.55 |  | 2,401,332 | 4.45 | 0.50 |
| **LTR** | 142,923,156 | 67.03 | 29.82 |  | 132,198,103 | 85.02 | 27.58 |  | 113,814,744 | 57.84 | 23.75 |  | 47,025,651 | 87.08 | 9.81 |
| **RC** | 18,558,089 | 8.70 | 3.87 |  | 7,897,317 | 5.08 | 1.65 |  | 17,490,177 | 8.89 | 3.65 |  | 70,989 | 0.13 | 0.02 |
| **rRNA** | 50,106 | 0.02 | 0.01 |  | - | - | - |  | 50,106 | 0.03 | 0.01 |  | - | - | - |
| **SINE** | 1,305,603 | 0.61 | 0.27 |  | 7,279 | 0.00 | 0.00 |  | 1,298,324 | 0.66 | 0.27 |  | - | - | - |
| **Satellite** | 183,110 | 0.09 | 0.04 |  | 182,902 | 0.12 | 0.04 |  | 208 | 0.00 | 0.00 |  | - | - | - |
| **Unknown** | 46,498,248 | 21.81 | 9.70 |  | 27,784 | 0.02 | 0.01 |  | 46,471,369 | 23.62 | 9.70 |  | - | - | - |
| **Other** | 7,183 | 0.00 | 0.00 |  | 7,183 | 0.00 | 0.00 |  | - | - | - |  | - | - | - |
| **Total^1^** | 213,236,753 | 100 | 44.49 |  | 155,496,679 | 100 | 32.44 |  | 196,787,079 | 100 | 41.06 |  | 54,001,706 | 100 | 11.27 |

^1^Total repeat regions were identified combining all the repeats identified. As there are some overlaps between different methods, the total region is shorter than the sum of repeats identified by all methods.

**Table S8: Statistics of predicted protein-coding genes in the** ***P. pruinosa* genome.**

| **Gene Set** | | **Number** | **Average Transcript Length**  **(bp)** | **Average CDS Length**  **(bp)** | **Average Exon per Gene** | **Average Exon Length (bp)** | **Average Intron Length (bp)** |
| --- | --- | --- | --- | --- | --- | --- | --- |
| **Homolog** | *A. thaliana* | 22,634 | 3560.59 | 1248.68 | 4.29 | 291.13 | 702.91 |
|  | *C. papaya* | 34,441 | 2370.55 | 1000.08 | 3.31 | 302.20 | 593.46 |
|  | *P. trichocarpa* | 37,820 | 3058.24 | 1144.81 | 4.10 | 279.21 | 617.22 |
|  | *R. communis* | 29,038 | 2966.00 | 1199.52 | 4.10 | 292.65 | 570.05 |
|  | *P. euphratica* | 40,077 | 3079.91 | 1084.02 | 3.95 | 274.42 | 676.51 |
|  | *E. grandis* | 26,529 | 3845.52 | 1263.50 | 4.23 | 298.75 | 799.58 |
| ***De novo*** | GENESCAN | 32,887 | 6374.18 | 1202.34 | 5.70 | 210.80 | 1099.56 |
|  | AUGUSTUS | 35,596 | 3208.19 | 1212.88 | 5.39 | 224.89 | 454.18 |
| **RNA-Seq** | PASA | 41,843 | 2764.88 | 1040.01 | 4.74 | 219.35 | 461.04 |
| **EVM** |  | **35,131** | **3703.40** | **1224.38** | **5.41** | **226.27** | **561.98** |

**Table S9: Functional annotation of predicted genes for *P. pruinosa.***

|  |  | **Number** | **Percent (%)** |
| --- | --- | --- | --- |
| **Total** |  | 35,131 | 100.00 |
| **Annotated** | GO | 22,361 | 63.64 |
|  | KEGG | 11,746 | 33.43 |
|  | SwissProt | 30,783 | 87.62 |
|  | TrEMBL | 30,690 | 87.34 |
|  | InterPro | 26,505 | 75.43 |
| **Unannotated** |  | 4,193 | 11.94 |

**Table S10: Summary of syntenic blocks between *P. pruinosa* and *P. euphratica* identified using MCScanX.**

| **Species** | **Total genes** | **Syntenic genes** | **Genes in blocks** | **Block length (bp)** |
| --- | --- | --- | --- | --- |
| ***P. pruinosa*** | 35,131 | 22,714 | 29,015 | 290,081,475 |
| ***P. euphratica*** | 34,279 | 23,390 | 27,804 | 293,357,943 |

**Table S11: Top 10 GO categories (biological process and molecular function) displaying the highest Ka/Ks ratios between *P. pruinosa* and *P. euphratica*.**

| **GO categories within biological process** | | **Number of orthologs** | **Ka/Ks** |
| --- | --- | --- | --- |
| GO:0030001 | metal ion transport | 45 | 0.55 |
| GO:0048507 | meristem development | 15 | 0.53 |
| GO:0009888 | tissue development | 18 | 0.50 |
| GO:0010468 | regulation of gene expression | 19 | 0.50 |
| GO:0006626 | protein targeting to mitochondrion | 41 | 0.49 |
| GO:0006351 | transcription, DNA-dependent | 60 | 0.49 |
| GO:0030003 | cellular cation homeostasis | 27 | 0.47 |
| GO:0006855 | drug transmembrane transport | 46 | 0.47 |
| GO:0050826 | response to freezing | 41 | 0.46 |
| GO:0050896 | response to stimulus | 44 | 0.45 |
| **GO categories within molecular function** | | **Number of orthologs** | **Ka/Ks** |
| GO:0043531 | ADP binding | 36 | 0.61 |
| GO:0015297 | antiporter activity | 28 | 0.55 |
| GO:0031072 | heat shock protein binding | 23 | 0.54 |
| GO:0003755 | cis-trans isomerase activity | 48 | 0.54 |
| GO:0004784 | superoxide dismutase activity | 16 | 0.53 |
| GO:0005096 | GTPase activator activity | 15 | 0.51 |
| GO:0016765 | transferase activity | 35 | 0.50 |
| GO:0030247 | polysaccharide binding | 25 | 0.48 |
| GO:0016706 | oxidoreductase activity | 35 | 0.44 |
| GO:0009055 | electron carrier activity | 91 | 0.44 |

**Table S12: Summary of gene family clustering.** The genes from eleven sequenced plant genomes were collected and aligned to each other using BLASTP. Information on protein sequence pairwise similarity was used as distance to cluster genes.

| **Species** | **Total**  **genes** | **Genes in families** | **Family** | **Unclustered genes** | **Unique families** | **Genes per family** |
| --- | --- | --- | --- | --- | --- | --- |
| *V. vinifera* | 26,346 | 19,422 | 12,721 | 6,924 | 642 | 1.53 |
| *E. grandis* | 36,349 | 28,785 | 13,584 | 7,564 | 826 | 2.12 |
| *A. thaliana* | 27,416 | 23,315 | 12,850 | 4,101 | 710 | 1.81 |
| *C. papaya* | 27,751 | 19,615 | 13,093 | 8,136 | 576 | 1.50 |
| *F. vesca* | 32,831 | 25,062 | 13,612 | 7,769 | 1,390 | 1.84 |
| *C. sativus* | 21,503 | 17,781 | 12,458 | 3,722 | 212 | 1.43 |
| *R. communis* | 31,221 | 20,639 | 14,630 | 10,582 | 742 | 1.41 |
| *S. suchowensis* | 26,599 | 23,513 | 14,436 | 3,086 | 97 | 1.63 |
| *P. trichocarpa* | 41,335 | 33,907 | 18,015 | 7,428 | 410 | 1.88 |
| *P. euphratica* | 34,279 | 31,038 | 17,871 | 3,241 | 149 | 1.74 |
| *P. pruinosa* | 35,131 | 28,773 | 17,592 | 6,358 | 224 | 1.64 |

**Table S13. Analysis of *P. pruinosa* species-specific genes.**

| **Species** | **Total gene number** | **Genes in families** | **Family number** | **Species-specific genes** | | | | |
| --- | --- | --- | --- | --- | --- | --- | --- | --- |
|  |  |  |  | **Unclustered genes** | **Unique families** | **Total No.** | **With functional support** | **With expression support^1^** |
| *P. pruinosa* | 35,131 | 28,773 | 17,592 | 6,358 | 224 | 7,020 | 3,302 | 1,760 |

^1^RPKM > 0.5 was used to detect the genes supported by expression data.

**Table S14: GO enrichment analysis of species-specific genes in the *P. pruinosa* genome.** For each GO subcategory, a 2 × 2 contingency table was constructed by recording the numbers of genes included or not included in a category of ‘genome background’ genes and species-specific genes. Two-tailed Fisher’s exact test was used to calculate statistical significance.

| **GO ID** | **Description** | **Taxonomy** | **Number**  **of genes** | **P-value** |
| --- | --- | --- | --- | --- |
| GO:0001071 | Transcription factor activity | MF | 27 | 7.06E-10 |
| GO:0004871 | Signal transducer activity | MF | 12 | 4.00E-04 |
| GO:0008270 | Zinc ion binding | MF | 74 | 4.00E-04 |
| GO:0005215 | Transporter activity | MF | 73 | 1.10E-03 |
| GO:0046148 | Pigment biosynthetic process | BP | 10 | 1.10E-02 |
| GO:0009651 | Response to salt stress | BP | 12 | 1.22E-02 |
| GO:0003006 | Developmental process involved in reproduction | BP | 56 | 1.25E-02 |
| GO:0015075 | Ion transmembrane transporter activity | MF | 30 | 1.25E-02 |
| GO:0002376 | Immune system process | BP | 18 | 1.70E-02 |
| GO:0030154 | Cell differentiation | BP | 21 | 1.87E-02 |
| GO:0048583 | Regulation of response to stimulus | BP | 32 | 2.46E-02 |
| GO:0006811 | Ion transport | BP | 64 | 3.00E-02 |
| GO:0016628 | Oxidoreductase activity | MF | 22 | 3.02E-02 |
| GO:0048544 | Recognition of pollen | BP | 19 | 4.90E-02 |

**Table S15: GO enrichment analysis of expanded gene families in the *P. pruinosa* genome.** For each GO subcategory, a 2 × 2 contingency table was constructed by recording the numbers of genes included or not included in a category of ‘genome background’ genes and expanded genes. Two-tailed Fisher’s exact test was used to calculate statistical significance.

| **GO ID** | **Description** | **Taxonomy** | **Number**  **of genes** | **P-value** |
| --- | --- | --- | --- | --- |
| GO:0015833 | Peptide transport | BP | 18 | 1.02E-08 |
| GO:0046527 | Glucosyltransferase activity | MF | 33 | 6.03E-08 |
| GO:0030570 | Pectate lyase activity | MF | 13 | 8.73E-08 |
| GO:0006334 | Nucleosome assembly | BP | 13 | 1.06E-06 |
| GO:0004185 | Serine-type carboxypeptidase activity | MF | 13 | 5.07E-06 |
| GO:0043531 | ADP binding | MF | 32 | 1.06E-05 |
| GO:0006869 | Lipid transport | BP | 20 | 1.38E-05 |
| GO:0009892 | Negative regulation of metabolic process | BP | 45 | 3.45E-05 |
| GO:0005261 | Cation channel activity | MF | 11 | 8.23E-05 |
| GO:0061134 | Peptidase regulator activity | MF | 13 | 9.15E-05 |
| GO:0009653 | Anatomical structure morphogenesis | BP | 16 | 1.30E-03 |
| GO:0001071 | Transcription factor activity | MF | 28 | 1.80E-03 |
| GO:0006813 | Potassium ion transport | BP | 12 | 1.50E-02 |
| GO:0030154 | Cell differentiation | BP | 12 | 2.10E-02 |
| GO:0015297 | Antiporter activity | MF | 14 | 2.50E-02 |
| GO:0033554 | Cellular response to stress | BP | 20 | 3.30E-02 |
| GO:0016709 | Oxidoreductase activity | MF | 15 | 3.40E-02 |
| GO:0045859 | Regulation of protein kinase activity | BP | 18 | 4.20E-02 |

**Additional Figures**


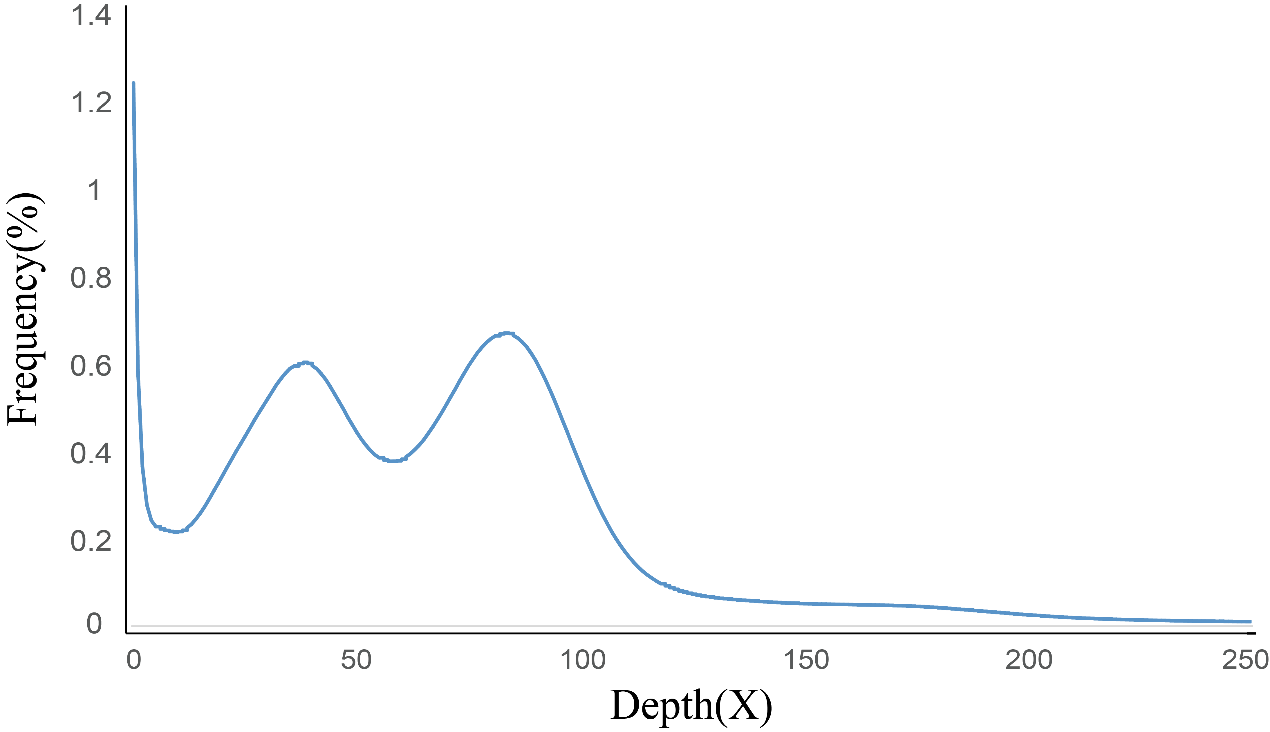


**Figure S1: 17-mer analysis for** ***P. pruinosa* genome based on clean reads from paired-end libraries.**

**
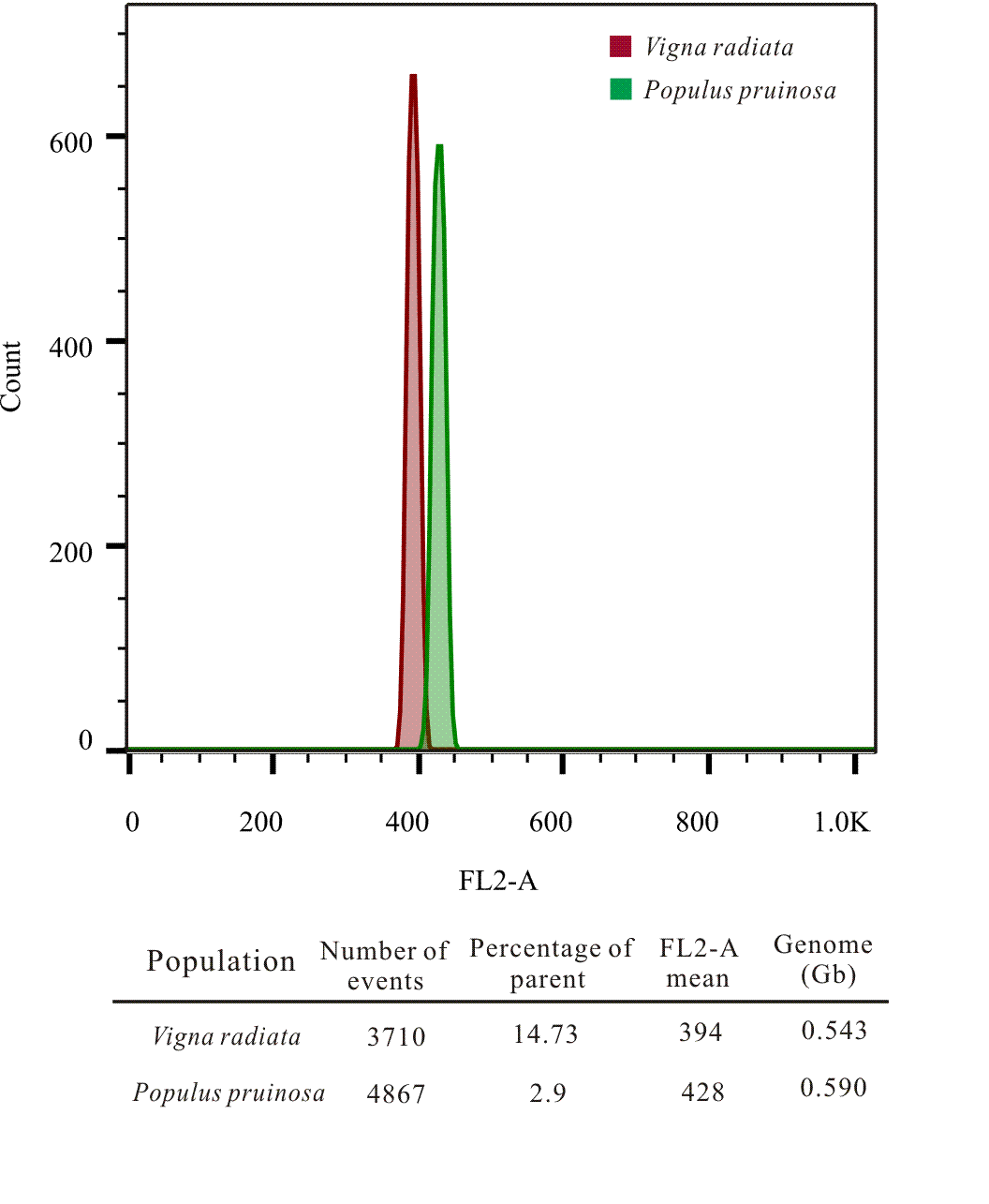
**

**Figure S2:** **Flow cytometry estimate of the *P. pruinosa* genome size compared to reference standard of *Vigna radiate* (543Mb).**

**
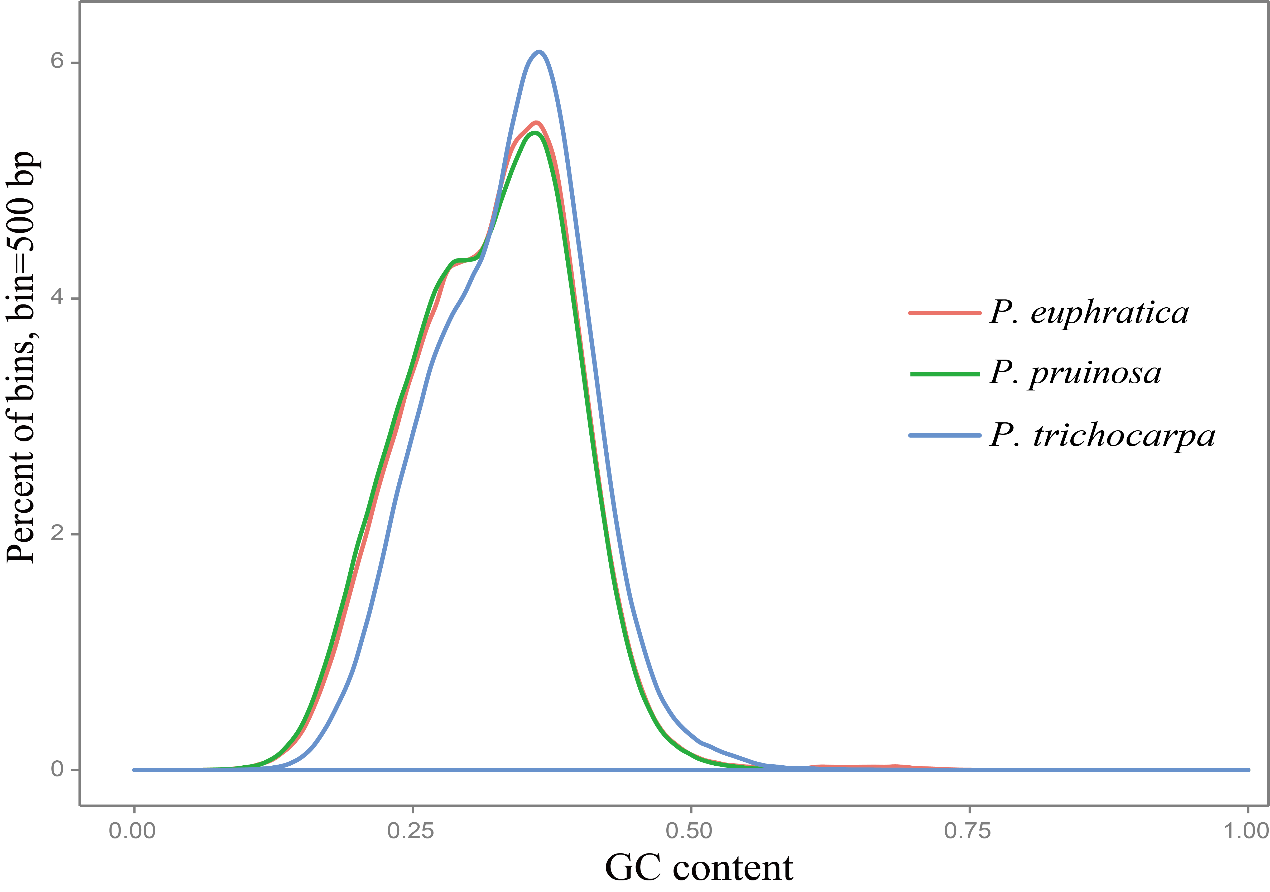
**

**Figure S3: GC content distribution for the genomes of *P. pruinosa* and related poplar species, established by 500 bp non-overlapping sliding windows.**

**
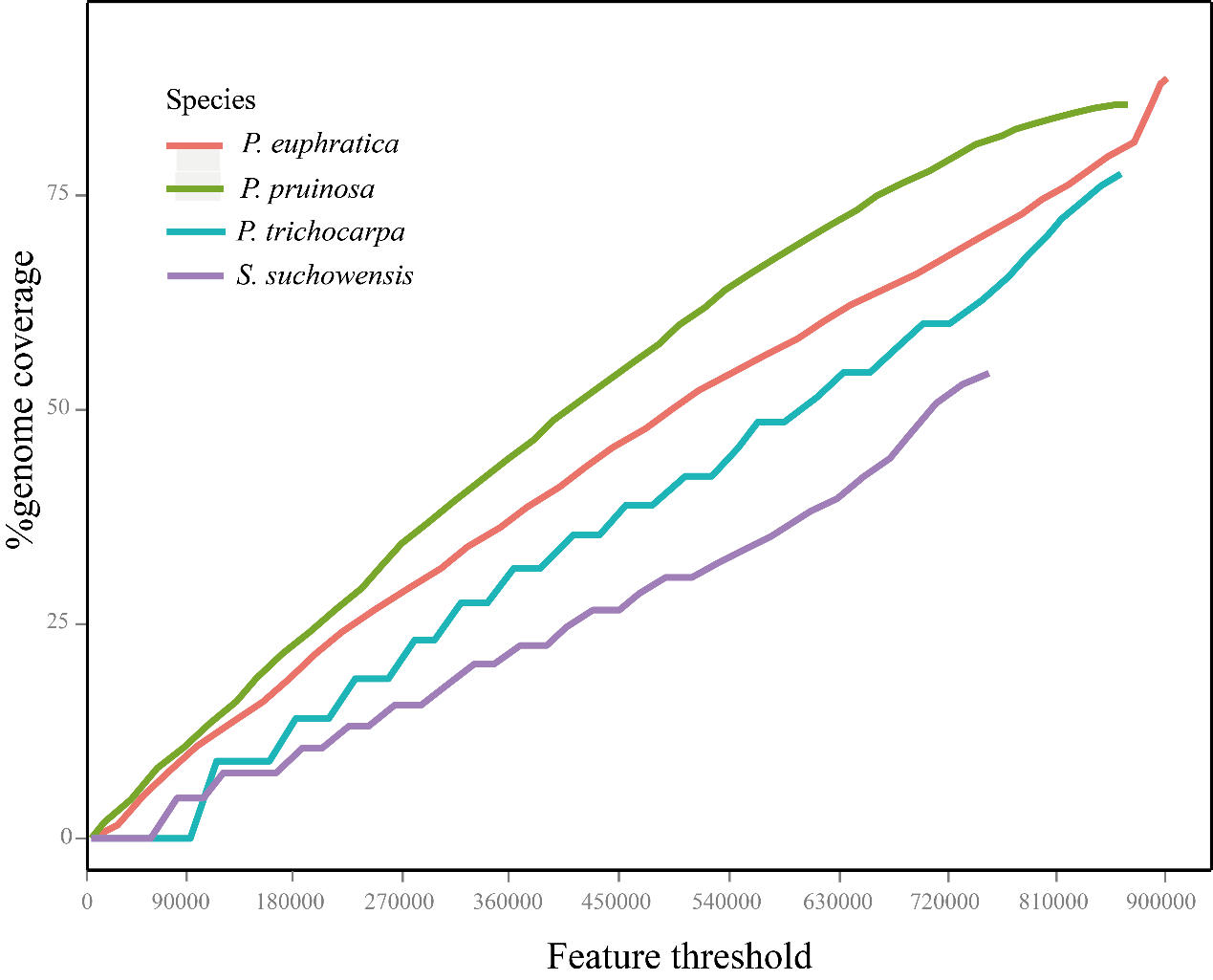
**

**Figure S4: FRCurve of four genome assemblies.** The FRCurve were calculated by the software FRC_align (<https://github.com/vezzi/FRC_align>).

**
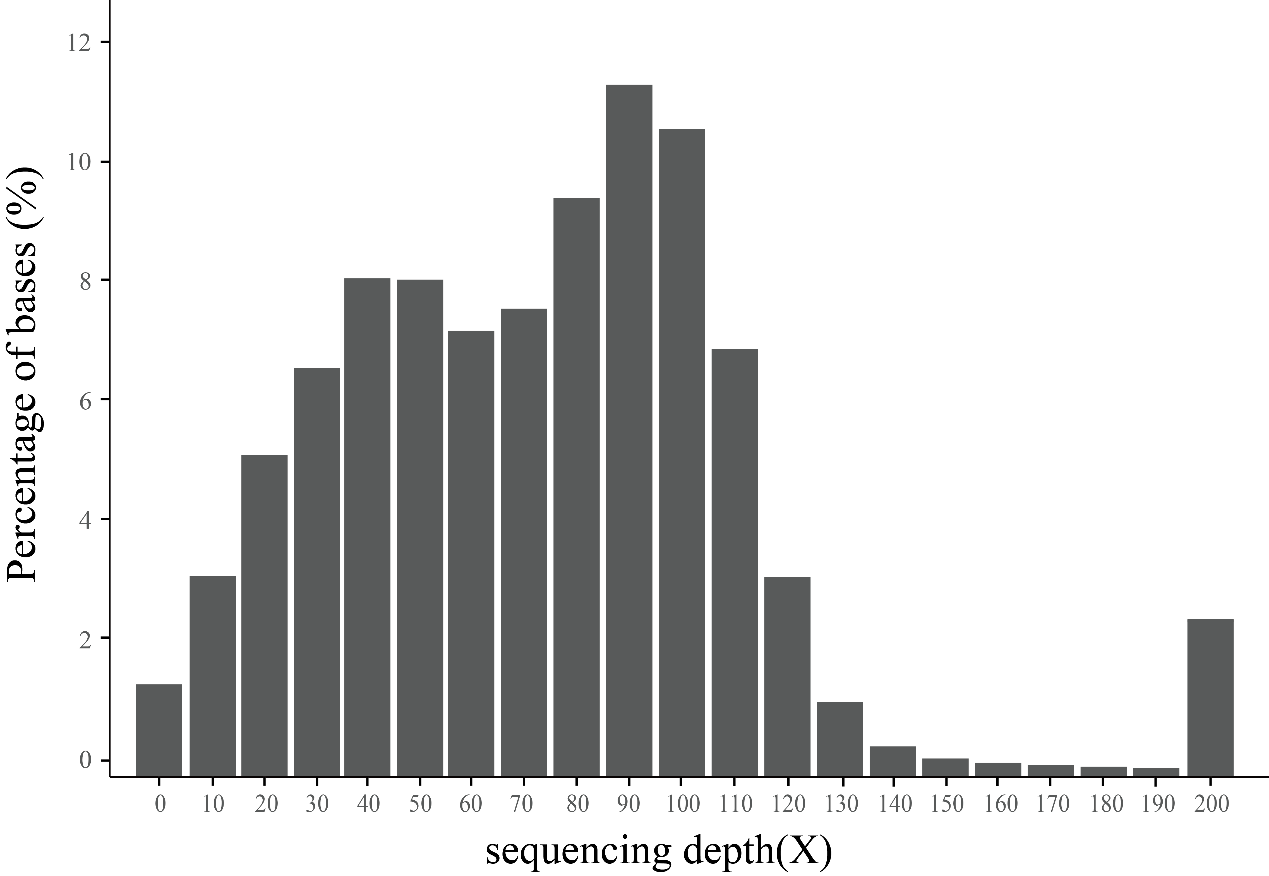
**

**Figure S5: Sequencing depth distribution for the *P. pruinosa* genome.** The sequencing reads from paired-end libraries were realigned onto the assembly using the BWA software. The sequencing depth of each base was calculated and plotted.


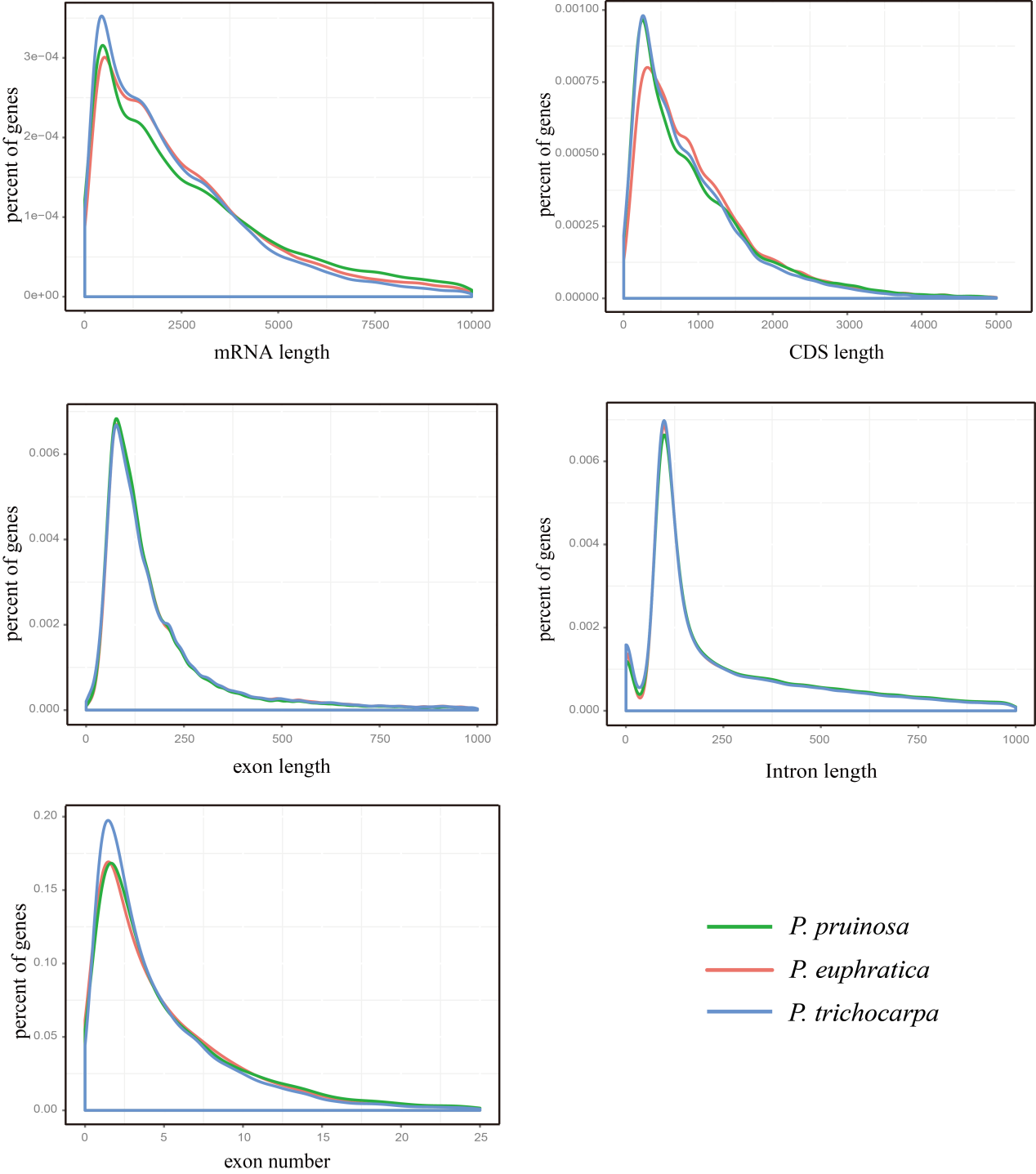


**Figure S6: Comparison of mRNA length (A), CDS length (B), Exon length (C), Intron length (D), and Exon number per gene (E) in** ***P. pruinosa* and in related poplar species.**


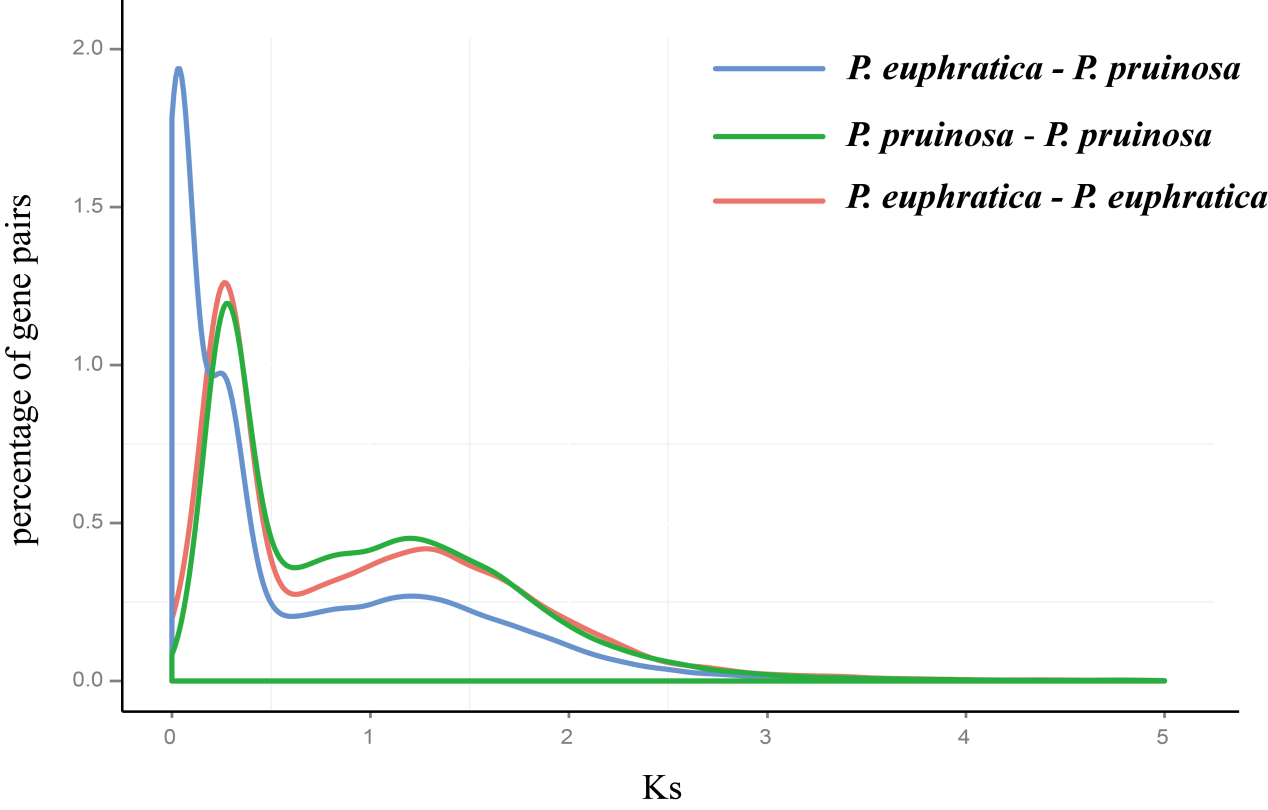


**Figure S7: Genome duplication in *Populus* genomes as revealed by Ks analyses.**


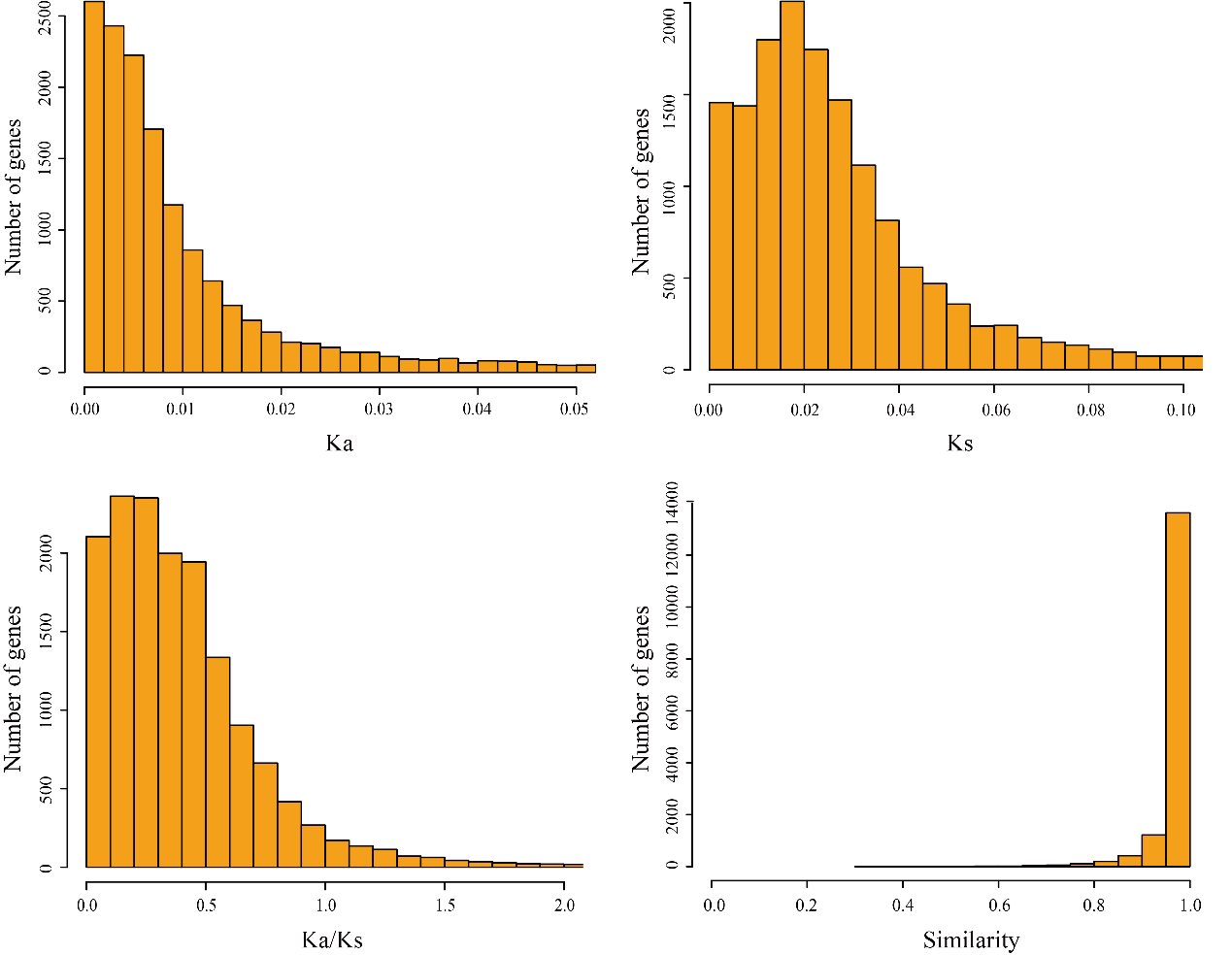


**Figure S8: Distribution of Ka, Ks, Ka/Ks and protein similarity in 1:1 *P. pruinosa-P. euphratica* orthologs within syntenic blocks.** To calculate the percent protein similarity between two sequences, the number of identical residues was divided by the total number of alignment positions.


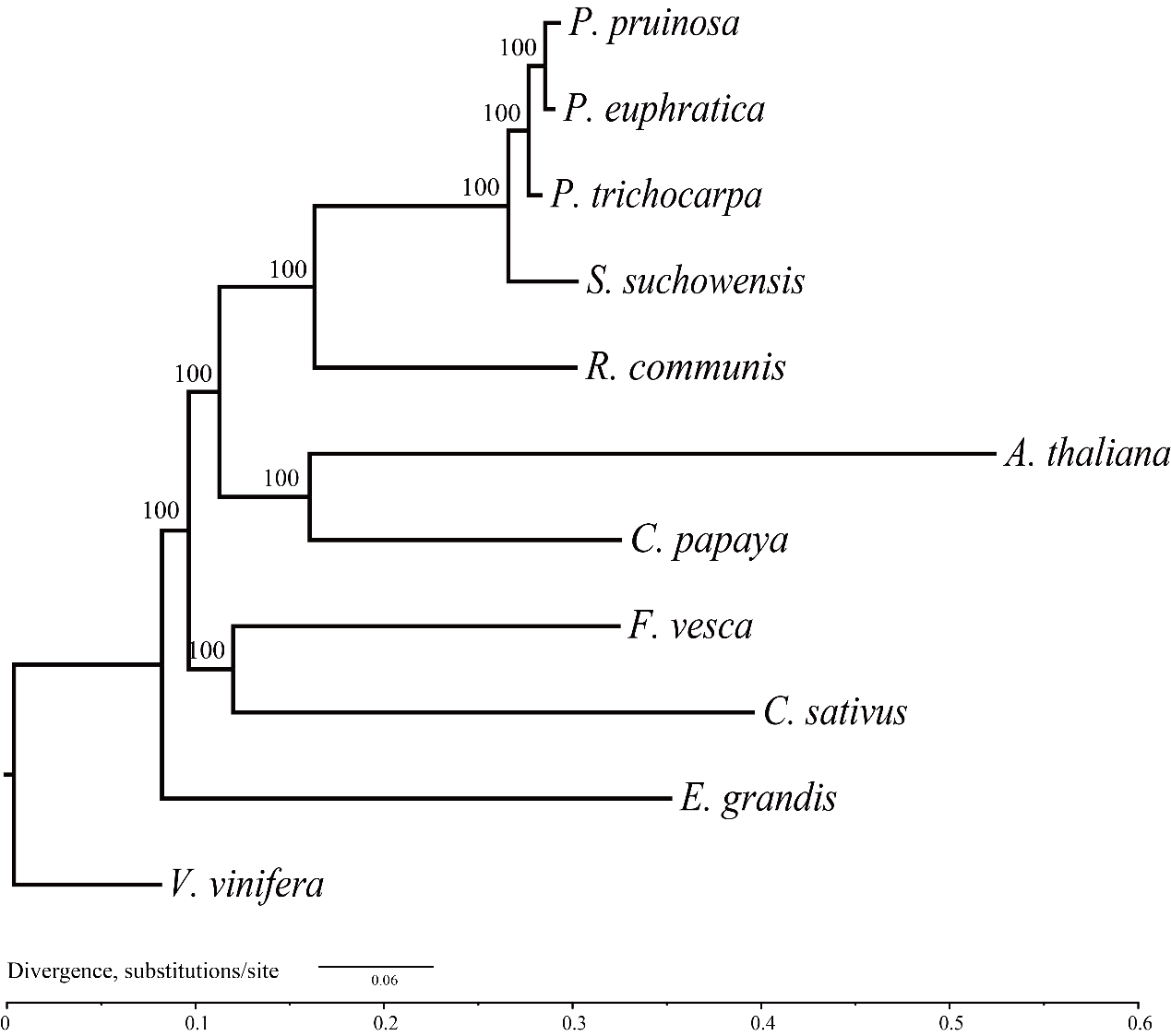


**Figure S9: Phylogenetic relationships of *P. pruinosa* and 10 other plant species.** The numbers on the nodes represent the bootstrap values.


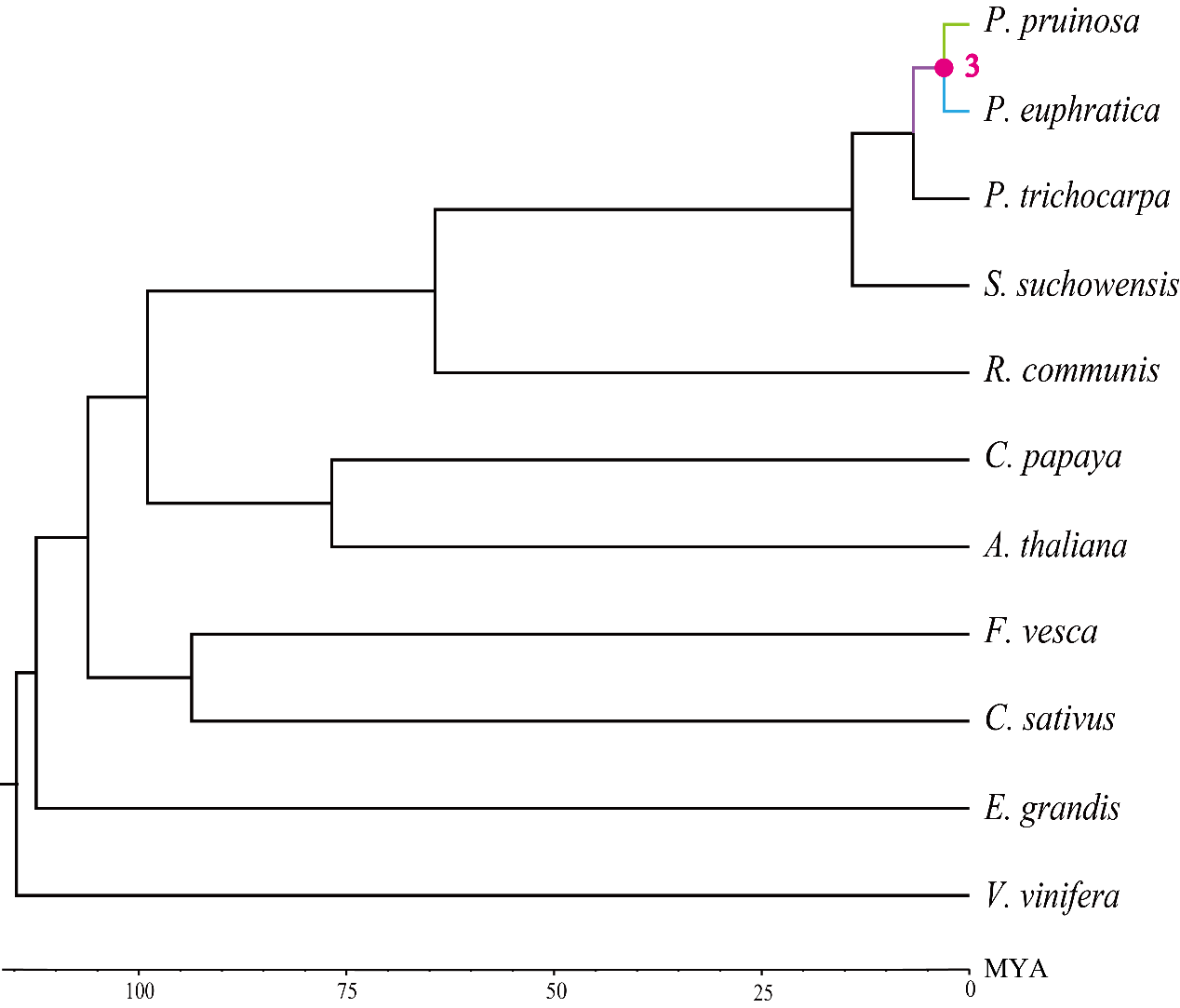


**Figure S10: Estimation of divergence time between *P. pruinosa* and *P. euphratica* using phylogenetic analysis.** The number on the node represent the estimated divergence time from present (million years ago, Mya). The calibration times for divergence between *A. thaliana* and *C. papaya* (54-90 Mya), *A. thaliana* and *R. communis* (95-109 Mya), *V. vinifera* and *A. thaliana* (106-119 Mya), were obtained from the TimeTree database (http://www.timetree.org/).


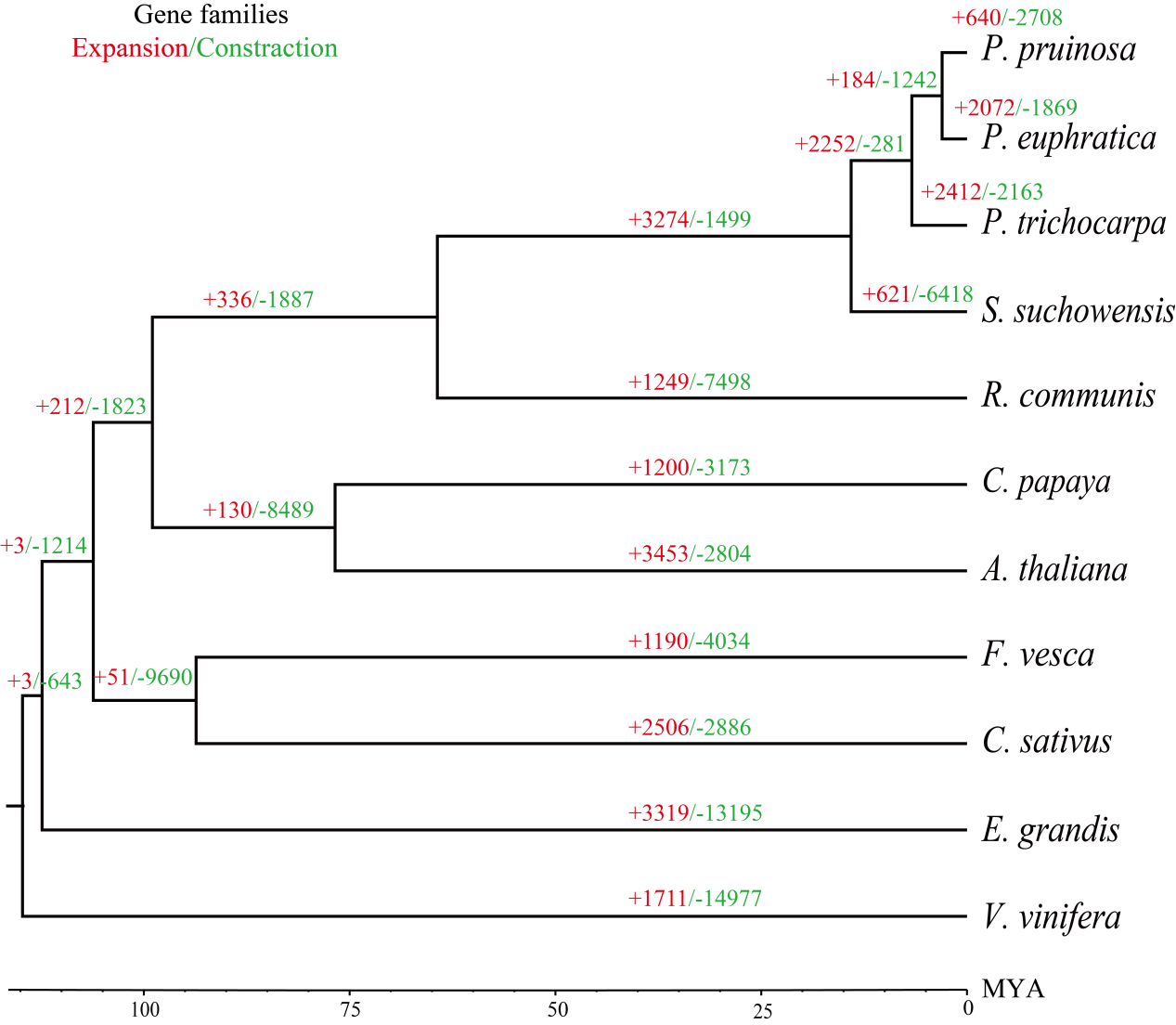


**Figure S11: Dynamic evolution of orthologous gene families.** Gene family expansion events are shown in red and gene family contraction events in green.
