# Supplementary material for: The draft genome sequence of a desert tree Populus pruinosa
Source: Gigascience. 2017 Aug 8;6(9):1–7. doi: 10.1093/gigascience/gix075 (PMC5603765; doi:10.1093/gigascience/gix075)
Supplement: Response-to-Reviewer-Comments_Original-Submission.pdf [file gix075_Response-to-Reviewer-Comments_Original-Submission.pdf]

Author's Response To Reviewer Comments

Reviewer #1: In this manuscript, Yang et al presents the draft genome sequence of a desert Poplar species, Populus pruinosa. This is the second of three poplar species being sequenced from the Turanga section of poplar, which brings the total number of poplar genomes sequenced to three. This offers a unique opportunity for comparative genomics within the poplar family.
The Populus pruinosa genome was assembled from 60 GB of Illumina paired end and mate pair data (107X coverage of the genome) to (unknown number of) contigs with an N50 of 14 kb and 78960 scaffolds with an N50 of 698.5 kb. No draft assembly is perfect, but long read data will definitely contribute to a higher quality assembly.
The authors then went and described the annotation of the genome, as well as an evolutionary analyses between P. pruinosa and P. euphratica. The authors identified genes unique to the P. pruinosa genome using ortholog clustering methods, and identified a set of genes which shows adaptive divergence between the P. euphratica and P. pruinosa.

Reply: We appreciate the reviewer's positive comments on this work. We have added the information of contig and scaffold number in Table S3.

Please see below for specific criticisms of the manuscript:

1) Background - lines 45-47: The statement "We recovered an unexpectedly large number of genetic variations between these two sister species" needs better support in the manuscript. SNP analyses were done with the short reads aligned to the P. pruinosa genome (Genome assembly - lines 19-36). You do, however, mention a large number of species specific genes present within the P. pruinosa assembly that do not cluster with 10 other plant species (Table S12). This is a very important statement in the paper.

Reply: We have improved the statements according to the suggestions of the reviewer.

2) Samples and Sequencing: The RNA sampling and preparation is not described.

Reply: We have added the related description in the revised manuscript.

3) Samples and Sequencing: lines 9-12: Parameters for low quality base trimming not mentioned. If this is not stringent enough, it would explain the high heteroxygosity found.

Reply: We have added the detailed criterion for quality checking and read filtering in the revised manuscript. The version numbers and command line arguments for all programs used in this work are also released in the GigaScience database.

4) Table S2 not required, can be described in text.

Reply: Done.

5) Genome Assembly: lines 34-36. What k-mers were used for the Platanus assembly.

Reply: The default parameter '-k 32' was used for the Platanus assembly.

6) Genome Assembly: lines 47-51. What portion of the scaffolds contains gaps?

Reply: The information was added in both the main text and Table S3.

7) Genome Assembly lines 25-32: GATK parameters for SNP calling missing

Reply: The parameters for SNP calling and scripts for quality filtering were released in the GigaScience database.

8) Genome Assembly lines 32-36: Heterozygosity levels for P. pruinosa are double that of P. euphratica (0.86% vs 0.49%). Is the SNP calling parameters stringent enough?

Reply: We have discarded relevant descriptions about the comparison of heterozygosity levels between these two species, since the different software and parameters maybe biased the results as suggested by the second reviewer.

9) Repeat Annotation: lines 59 -1: Clarify how you reached the 45% repetitive elements in the genome. From Table S6 it looks like an addition of found elements, but surely there can be an overlap between different alorithms.

Reply: We have added the statement in both the main text and relevant Tables.

10) Gene Annotation: lines 16-19. Five plant species were used for genome annotation. The other tree genome, Eucalyptus grandis, is missing from the list.

Reply: We have added the genome of Eucalyptus grandis for the gene prediction of our assembly, and re-done almost all the subsequent analysis in our revised manuscript.

11) Evolutionary Analysis: lines 9-20: A figure showing the syntheny blocks and/or alignments will highlight the co-linearity of the two genomes

Reply: We added a figure in the main text as suggested by the reviewer.

12) Evolutionaly Analysis: lines 1-5: Ten plant species were used for the evolutionary analyses. E. grandis should perhaps be included here?

Reply: Done.

13) Evolutionary analyses: lines 10-15: "607 genes being specific to P. pruinosa". A file containing the annotation of these genes will aid in figuring out what these genes are.

Reply: We have released this table in the GigaScience database.

14) Evolutionary analysis: lines 15-17: What constitutes RNA support?

Reply: We have added the criterion (RPKM > 0.5) in the main text and table S13.

15) Evolutionary analysis: lines 58-60: Remove "significant" from the sentence "… a significant number of genes show…". Significance can not be shown here.

Reply: Done.

I am satisfied that the availability of the P. pruinosa genome will contribute to the study of the evolutionary history of Poplar species. I do, however request that the authors consider the criticisms highlighted above. I also request that the alignment files (.bam) and .gff files used for SNP identification and annotation is made available through the website listed in the manuscript. This would include the evident files for the RNA-Seq work.

Reply: We are grateful for these positive comments and have addressed all of the points raised by the reviewer. All of the data described in this manuscript are available in the GigaScience database.

Reviewer #2: Please use continuous line numbers when you submit manuscripts. It is infuriating to have to refer to page and line number, especially as page numbers are not even included. I have stated line numbers and I leave it up to the authors to find the relevant page.

Reply: We are very sorry about this. We have used continuous line numbers in our revised manuscript.

While the presented genome assembly and annotation appear to have been conducted sensibly, the presented methods are inadequate at present to enable a meaningful assessment of this. There is value in the presented genome assembly compared to the available P. euphratica assembly in that the presented draft is more contiguous, however this requires validation. The included evolutionary analyses are potentially interesting but are limited in scope, contain no population level data and are entire descriptive. Such analyses are also frequently problematic when based on a draft genome assembly, and this problem is exacerbated here by use of two draft assemblies, with no assessment or apparent consideration for how this may influence to results obtained. As the associated web resource is currently non-functional and of limited scope (for example, there appears to be no link to P. trichocarpa or other species), the draft assembly is of limited potential use to the wider community.

Reply: We appreciate the reviewer’s comments on our work. We reworded the relevant part according to the suggestions of the referee and found all the suggestions to be highly useful for improving our manuscript.

L5-7. It is absolutely not true that little is known about the evolutionary genomics of Populus species. There is an existing body of literature relating to this and the evolutionary genomics and population genetics of various Populus species have been the focus of many past and recent

studies. Of note, and also of relevance to the following sentence, the authors should be aware of the recent paper discussing the pan genome of P. nigra (https://doi.org/10.1093/molbev/msw161). There are also extensive genomics resources available for Populus. In short, this is a completely inadequate coverage of the extensive Populus literature that subsequently creates a false impression of novelty for the presented resource.

Reply: We are very sorry about the statements. We have corrected these descriptions in the revised manuscript.

L14. What is your evidence for the statement that these two genomes do not cover the diversity observed? If this is an observation then state the source of this.

Reply: We have discarded these descriptions in the revised manuscript.

L15 Please include the authority for at first use of the species.

Reply: Done.

L26. Habitats are not specialized. Species living within habitats may evolve specialised adaptations to survive in those habitats.

Reply: We have changed the 'specialized' to 'distinct'.

L45 Unexpected to whom? There is nothing unexpected about the large number of variants identified, with similar results reported for other Populus species.

Reply: We have reworded this unclear statement in the revised manuscript.

The entire Data description section is wholly inadequate. Methods are not detailed, software versions and parameters used are not given. As such it would be completely impossible to recreate the presented work. For example, my group has experience using the Platanus assembler with Illumina data from highly heterozygous species and we would love to know how you ran the assembly to get such high contiguity.

Reply: We have added the detailed description of the methods and the software versions in the revised manuscript. We also released our assembly pipeline, assessment pipeline and command line arguments for all the programs in the GigaScience database, which might be useful for further studies.

Please give proper details about how the DNA was extracted, the kit versions used for the Illumina sequencing (this matters as different kit versions have different GC bias) and how the large-insert libraries were constructed.

Reply: Done.

L9. More detail is needed to understand how contaminated reads were removed. How were these

classified? Was there any subsequent checking of the assembly to remove contaminant contigs?

Reply: We sincerely apologize for this misunderstanding which could be fully avoided if we had provided more details before. The detailed information of quality checking and read filtering were added in the revised manuscript.

L11. Be consistent in the terminology used for cleaned reads. They are also referred to as qualified reads, which I would suggest is not the most suitable or standard term.

Reply: We have changed the ‘qualified reads’ to ‘clean reads’.

The use of kmer based methods to estimate genome size is notoriously error prone. It would have been preferable to see a range of kmers used to indicate likely error in genome size estimation. Even better would have been wet lab confirmation of genome size using flow cytometry. At the least, the use of the selected kmer size should be justified. It is also rather strange that the only parameters stated for any software are for KmerFreq_AR.

Reply: We are very much in agreement with the review’s views and doubts. In the revised manuscript, we have discarded the kmer-based methods and instead used flow cytometry to estimate the genome size of P. pruinosa.

There is no attempt to validate or assess the quality of the assembly. This could have been done using one of the available tools (such as FRC analysis methods), for example.

Reply: We have added the FRC analysis to evaluate the quality of our genome assembly.

L2. CEGMA and BUSCO are redundant and only one should be used. As detailed in the BUSCO publication, this is a replacement for CEGMA, with CEGMA no longer being under active development. I would also argue that a far more comprehensive analysis of gene space completeness could have been performed using the two available Populus genome annotations, as BUSCO is still rather limited. There are similarly more comprehensive such datasets available based on the PLAZA genome resource from Yves Van de Peer's group, where largely single copy genes have been identified across plant species. Please also refer to gene space completeness rather than genome completeness, as assessment of genes does not indicate how complete the entire assembly is.

Reply: We have discarded the relevant results of CEGMA. According to the suggestions of the referee, we also used the protein-coding genes predicted in the P. euphratica and P. trichocarpa genomes to evaluate the gene space completeness of our assembly.

L19-23. This could have been analysed more comprehensively. For example, it would have been highly informative to see an assessment of how many split haplotypes are represented in the assembly. This also has important implications for interpreting the presented gene family and synteny analyses.

Reply: It is well known that de novo genome assembly of highly heterozygous species is a

substantial challenge for whole-genome shotgun reads. Two divergent haplotypes will increase the complexity of the de Bruijn graph structure and lead to the presence of split haplotypes in the assembly as raised by the reviewer. Examining these split haplotypes is a critical aspect to evaluate the accuracy and quality of the assembly. But we found that there is no effective tools or methods to do that at least in our knowledge, after searching and reading a lot of literatures. However, we believe that it is not an important issue in our assembly and subsequently evolutionary analysis:
1) The P. pruinosa genome was assembled by Platanus, a software specifically designed for the assembly of highly heterozygous genomes. Our assembly has a size smaller than the genome size estimated by flow cytometry and similar with other poplar species.
2) The length distributions of transcripts, coding sequences, exons and introns predicted in P. pruinosa were similar to other poplar species. BUSCO analysis revealed a comparable coverage ratios both for single-copy and duplicated genes among the genomes of P. pruinosa, P. euphratica and P. trichocarpa.
3) We found extensive 1:1 synteny relationships between our assembly and the genomes of P. euphratica and P. trichocarpa.

L27. To state that GATK was used and to give no further details of how is, to be frank, ridiculous. The resulting heterozygosity estimate is entirely dependent on the settings used to run GATK so it is impossible to form an opinion of whether this value has any meaning or not as presented. For example, how do the settings used compare to those used for the equivalent analysis in P. euphratica? Does this comparison hold any value whatsoever? To be meaningful, the extent of haplotype splitting in both assemblies would need to be assessed and taken into consideration as this has massive implications for SNP calling. As presented, I see no biological value in the stated comparison.

Reply: We are very much in agreement with the review’s comments. We have discarded relevant descriptions about the comparison of heterozygosity levels between the two species.

Gene annotation. I would have liked to see the logic of the presented annotation presented. For example, why was PASA used rather than using the available RNASeq support as input to Augustus? These various support evidence types are anyway then integrated using EVidenceModeler. Similarly, I would like to know to justification for selecting the species used for protein support. Some of these genome are not high quality. There is also some confusing method description in this part. For example at L19 I do not follow the description of how homologous genome sequences were aligned. Why were partial genes and small genes removed and was any assessment performed of whether these may represent genes that are fragmented within the assembly?

Reply: We are very sorry about the doubts because of our unclear statements. PASA is a genome annotation tool that exploits spliced alignments of expressed transcript sequences (RNA-seq support) to automatically model gene structures. We believe the results of PASA is very stringent to identify the protein coding genes in our assembly. Additionally, the partial genes and small genes were removed only for the resultant data sets of de novo prediction, because this method will produce more false positive results. We have added the genome of Eucalyptus grandis for gene annotation as suggested by the first reviewer, and reworded the relevant part in

the section of ‘Gene annotation’. The annotation pipeline was also released in the GigaScience database.

The presented evolutionary analysis has, I believe, some important limitations and caveats. The most serious of these is the use of two draft, fragmented genome assemblies - which is particularly the case for P. euphratica. Draft assemblies are notoriously problematic for gene family analyses and for inference of contracted or expanded gene families, for example. I would also have liked to see some comparison to other Populus species. For example, are the gene identified as rapidly evolving specific to the comparison of the two species considered or do these represent genes with generally relaxed selection constraint in common across a broader range of species? This section (and the subsequent gene family analyses) is also far too speculative and descriptive yet is the stated novelty of the presented resource.

Reply: We are very much in agreement with the review’s comments. However, all of our evolutionary analysis were based on the protein-coding sequences. Our previous assessment of the gene space completeness for P. pruinosa genome revealed that our assembly covered most of the protein-coding genes. So we believe that our present results are not seriously affected by the quality of the genome assemblies. It is interesting to distinguish the rapidly evolving genes specific for the two sister species from the generally relaxed selection constraint across Populus species. However, in this study, we focused only on the identification of the genes with high divergence between these two sister species to elucidate the genome-wide differentiation between them.

L31 I have struggled to find this information at TimeTree for the two Populus species. Is there really fossil evidence to calibrate the divergence time for these two species specifically?

Reply: We apologize for this misunderstanding caused by our unclear statements. The divergence time between the two Populus species was estimated by software MCMCTree, not obtained from the TimeTree database. We have corrected this statement in the revised manuscript.

Availability of the genome: I have tried a number of times to visit the detailed web resource. On all occasions the genome and synteny browsers were non-functional. It also appears that only P. euphratica is available at the included BLAST tool. As such, the web resource appears to be of no value or use at present.

Reply: We are very sorry about the doubts because of our limited web resource. We only provided a temporary websites for reviewers to visit our data. Our present work don’t involve the web resource for genome browsers and BLAST. Instead, we have released all the data described in this manuscript in the GigaScience database.

Many of the legends and details in the supplementary material are not adequate. For example, what is TRF? It is not clear whether any filter was applied for minimum contig/scaffold size when N50 etc statistics were calculated. Please ensure that all relevant details are given and that all abbreviations are defined.

Reply: We have updated these unclear statements according to the suggestions of the reviewer.

Reviewer #3: The Data note presents the genome assembly of Populus pruinosa - a poplar tree species that is adapted to the deserts of western China and neighboring regions. It is the third genome of the Populus genus to be sequenced, and others are being developed. Poplars are important forest tree species from ecological and economic standpoints throughout the Northern hemisphere; therefore, the development of genome resources for poplars will have wide ranging impacts. The development of several genome sequences enables comparative and evolutionary studies and, the analysis of a desert-adapted species helps to develop knowledge that is relevant for adaptation to climate change and for combating desertification.
This note describes the sequencing, assembly, annotation and evolutionary analysis of the genome. The methods are appropriate and the analyses are extensive. The data metrics indicate that the assembly is of high quality. The evolutionary analyses indicate that this resource is likely to lead to valuable developments in the understanding of adaptation and evolution in plants.

Reply: We are grateful for the positive comments of the reviewer.

The outcomes of several analyses are presented in the supplemental materials but why not include a composite figure presenting the key findings in the main paper? I feel it would be off interest to readers and strengthen the paper.

Reply: We have added a figure and a summary table in the main paper as suggested by the reviewer.

I verified that the data have been deposited at the Short read archive for 7 Illumina libraries. I tried searching the project website (Salinity Tolerant Poplar Database) for information on the assembly. I did find a link but I was unsuccessful at downloading. Unfortunately I attempted this over Wifi which may be the problem. Could the authors give more detail on what is available and in what format? Could the authors also post an update on the website describing the genome assembly and its release?

Reply: We sincerely apologize for this doubts caused by our limited and temporary web resource. Instead, we have released all of the data described in this manuscript in the GigaScience database. They will be public after the manuscript is accepted.

In the section 'Evolutionary analyses', the last sentence of the 1st paragraph states that the functional categories are probably related to the differences in the adaptations... Please explain the basis for the statement / hypothesis.

Reply: We have discarded this baseless statement in our revised manuscript.

In the next paragraph in the same section, the authors wrote that they identified 6,925 genes that are specific to P. pruinosa, which seems like a large number given what is known about gene

conservation in plants. The authors should explain on what basis is the number obtained. Is the result relative to other poplars or relative to all plants?

Reply: The number of P. pruinosa specific genes was obtained by gene family clustering analysis across 11 plant genome. Both the unclustered genes and the genes within P. pruinosa unique families are included. We have updated this information in Table S13.

In the last sentence of the closing paragraph, the authors wrote that pan-analyses will be necessary in poplar but they do not explain why they believe this to be the case nor what it will achieve. I do not dispute the potential interest of the idea but I do feel it needs at least some explanation.

Reply: We have removed this statement in the revised manuscript.
