# Supplementary material for: The draft genome sequence of a desert tree Populus pruinosa
Source: Gigascience. 2017 Aug 8;6(9):1–7. doi: 10.1093/gigascience/gix075 (PMC5603765; doi:10.1093/gigascience/gix075)
Supplement: Response-to-Reviewer-Comments_Revision-2.pdf [file gix075_Response-to-Reviewer-Comments_Revision-2.pdf]

Author's Response To Reviewer Comments

Reviewer #1: I want to extend my thanks to the author for addressing the comments and concerns I had with the paper.

Reply: We are grateful for the positive comments of the reviewer.

Reviewer #2: The authors have improved the original manuscript and have addressed a number of my original concerns. However, some do remain. That being said, I am satisfied that the presented work is of sufficient scientific quality.
While it is good that the genome assembly and annotation is now made available, simply placing the fasta files on a FTP site does little service to the community. As the main benefit of the presented work is the potential for comparative analyses this is a great shame as the assembly is effectively of little utility to the community. I would strongly argue that the assembly and annotation should be placed at a central sequence resource such as NCBI or a Populus community resource.
Unfortunately I have found reading the author responses a rather frustrating experience. There are no references to line numbers to help the reviewers locate the changed parts of the manuscript and changes have not been indicated (for example using coloured text). Many of the original concerns were not addressed, simply being replied to with "We have removed this" and those removed components were what actually offered some of the most potentially interesting biology. As such the revised manuscript is now very much a data release note, although the increased focus and removal of speculative or over-extrapolated hypotheses has benefited the present manuscript. As a general comment and suggestion to the authors when writing future responses to reviewers, I at least find it far more useful to be directed to the relevant changes and to be provided with some discussion in response to comments from the reviewers.
Unfortunately there are still problems in the methods section - including the fact that readers are not directed to the relevant locations within the pipeline document provided on the GigaScience FTP site. For example (I am not detailing all similar such cases)

Reply: We appreciate the reviewer's positive comments on this work. We are very sorry for that no references to line numbers of the changed parts. We will consider this problem in the future responses to reviewers. All of the data produced by this manuscript have been released in the GigaScience database. The assembly sequences also have been deposited at NCBI, which will be released when the manuscript has been published. In this revised manuscript, we have further improved the statements of the pipeline document to provide the direct information of the generated files.

L66 What is 'the CTAB method'? I know what it is personally, but this is not how to write a proper methods section. The CTAB method is based on an original publication and there are many subsequent variations to that original method.

Reply: We have shared the CTAB method in the following protocols.io entry according to the suggestions of the Editor and added this information in the pipeline document provided on the GigaScience FTP:
https://www.protocols.io/view/ctab-dna-extraction-protocol-of-p-pruinosa-icdcas6

L67 Which Illumina protocol and what kit version?

Reply: We are very sorry about the lack of the detailed kit version of the Illumina protocol. We have sequenced the P. pruinosa genome at BGI-Shenzhen several years ago. They did not tell us the kit version and just provided the brief description as suggested in the manuscript (L67-L79). We also tried to ask the responsible manager but he did not remember. We are really sorry about this.

L75 I certainly would not be able to replicate this mate pair protocol given the provided details I appreciate that the online, main text version may need to be brief, but please then refer to a where full method details are provided.

Reply: We are very much in agreement with the review's comments. Because the P. pruinosa genome sequencing project has been launched several years ago, the protocol for constructing mate pair libraries was a little outdated.

L99 A two year old plant is arguable no longer a seedling. How were these plants grown? How were the samples obtained?

Reply: We have updated these statements according to the suggestions of the reviewer at L99-L101 in the revised manuscript.

L146 What other assemblies and better in what way? Presumably not better than the P. trichocarpa assembly, so which other assemblies are being used for this comparison?

Reply: We have updated this unclear statement at L145-L149 in the revised manuscript. It should be noted that the evaluation using FRC method of all genome assemblies were based on our P. pruinosa genome sequencing reads. A better FRCurve of our P. pruinosa genome assembly suggest that it is currently the best genome assembly for these reads (with best continuity) and it does not mean our P. pruinosa genome assembly is absolutely better than the other assemblies.

L152 20X is actually not so deep if this relates to the two collapsed haplotypes, as this represents only 10X per haplotype.

Reply: We only mapped the clean reads from the pair-end libraries to the reference genome to evaluate the accuracy of our assembly at the nucleotide level.
