# Supplementary material for: The draft genome sequence of a desert tree Populus pruinosa
Source: Gigascience. 2017 Aug 8;6(9):1–7. doi: 10.1093/gigascience/gix075 (PMC5603765; doi:10.1093/gigascience/gix075)
Supplement: Reviewer-1-Report-(Original-Submission).pdf [file gix075_Reviewer-1-Report-(Original-Submission).pdf]

Reviewer Report

Title: The draft genome sequence of a desert tree Populus pruinosa

Version: Original Submission **Date:** 2/7/2017

Reviewer name: Charles Hefer

Reviewer Comments to Author:

In this manuscript, Yang et al presents the draft genome sequence of a desert Poplar species, Populus pruinosa. This is the second of three poplar species being sequenced from the Turanga section of poplar, which brings the total number of poplar genomes sequenced to three. This offers an unique opportunity for comparative genomics within the poplar family.The Populus pruinosa genome was assembled from 60Gb of Illumina paired end and mate pair data (107X coverage of the genome) to (unknown number of) contigs with an N50 of 14kb and 78960 scaffolds with an N50 of 698.5kb . No draft assembly is perfect, but long read data will definitely contribute to a higher quality assembly.The authors then went and described the annotation of the genome, as well as a evolutionary analyses between P.pruinosa and P.eupharica. The authors identified genes unique to the P. pruinose genome using ortholog clustering methods, and identified a set of genes which shows adaptive divergence between the P. eupharica and P. pruinosa.Please see below for specific criticisms of the manuscript:1) Background - lines 45-47: The statement "We recovered an unexpectedly large number of genetic variations between these two sister species" needs better support in the manuscript. SNP analyses were done with the short reads aligned to the P. pruinosa genome (Genome assembly - lines 19-36). You do, however, mention a large number of species specific genes present within the P. pruinosa assembly that do not cluster with 10 other plant species (Table S12). This is a very important statement in the paper.2) Samples and Sequencing: The RNA sampling and preparation is not described.3) Samples and Sequencing: lines 9-12: Parameters for low quality base trimming not mentioned. If this is not stringent enough, it would explain the high heteroxygosity found.4) Table S2 not required, can be described in text.5) Genome Assembly: lines 34-36. What k-mers were used for the Platanus assembly.6) Genome Assembly: lines 47-51. What portion of the scaffolds contains gaps?7) Genome Assembly lines 25-32: GATK parameters for SNP calling missing8) Genome Assembly lines 32-36: Heterozygosity levels for P. pruinosa are double that of P. euphratica (0.86% vs 0.49%). Is the SNP calling parameters stringent enough?9) Repeat Annotation: lines 59 -1: Clarify how you reached the 45% repetitive elements in the genome. From Table S6 it looks like an addition of found elements, but surely there can be an overlap between different alorithms.10) Gene Annotation: lines 16-19. Five plant species were used for genome annotation. The other tree genome, Eucalyptus grandis, is missing from the list.11) Evolutionary Analysis: lines 9-20: A figure showing the syntheny blocks and/or alignments will highlight the co-linearity of the two genomes12) Evolutionaly Analysis: lines 1-5: Ten plant species were used for the evolutionary analyses. E. grandis should perhaps be included here?13) Evolutionary analyses: lines 10-15: "607 genes being specific to P. pruinose". A file containing the annotation of these genes will aid in figuring out what these genes are.14) Evolutionary analysis: lines 15-17: What constitutes RNA support?15) Evolutionary analysis: lines 58-60: Remove "significant" from the sentence "… a significant number of genes show…". Significance can not be shown

here.I am satisfied that the availability of the P. priunosa genome will contribute to the study of the evolutionary history of Poplar species. I do, however requist that the authors consider the critisisms highlighted above. I also request that the alignment files (.bam) and .gff files used for SNP identification and annotation is made availabe throught the website listed in the manuscript. This would include the evident files for the RNA-Seq work.

Level of Interest

Please indicate how interesting you found the manuscript: An article whose findings are important to those with closely related research interests

Quality of Written English

Please indicate the quality of language in the manuscript: Not suitable for publication unless extensively edited

Declaration of Competing Interests

Please complete a declaration of competing interests, considering the following questions:

- Have you in the past five years received reimbursements, fees, funding, or salary from an organisation that may in any way gain or lose financially from the publication of this manuscript, either now or in the future?
- Do you hold any stocks or shares in an organisation that may in any way gain or lose financially from the publication of this manuscript, either now or in the future?
- Do you hold or are you currently applying for any patents relating to the content of the manuscript?
- Have you received reimbursements, fees, funding, or salary from an organization that holds or has applied for patents relating to the content of the manuscript?
- Do you have any other financial competing interests?
- Do you have any non-financial competing interests in relation to this paper?

If you can answer no to all of the above, write 'I declare that I have no competing interests' below. If your reply is yes to any, please give details below.

I declare that I have no competing interests

I agree to the open peer review policy of the journal. I understand that my name will be included on my report to the authors and, if the manuscript is accepted for publication, my named report including any attachments I upload will be posted on the website along with the authors' responses. I agree for my report to be made available under an Open Access Creative Commons CC-BY license (http://creativecommons.org/licenses/by/4.0/). I understand that any comments which I do not wish to

be included in my named report can be included as confidential comments to the editors, which will not be published.

I agree to the open peer review policy of the journal
