# Supplementary material for: The draft genome sequence of a desert tree Populus pruinosa
Source: Gigascience. 2017 Aug 8;6(9):1–7. doi: 10.1093/gigascience/gix075 (PMC5603765; doi:10.1093/gigascience/gix075)
Supplement: Reviewer-2-Report-(Original-Submission).pdf [file gix075_Reviewer-2-Report-(Original-Submission).pdf]

Reviewer Report

Title: The draft genome sequence of a desert tree Populus pruinosa

Version: Original Submission **Date:** 2/8/2017

Reviewer name: Nathaniel Street

Reviewer Comments to Author:

Please use continuous line numbers when you submit manuscripts. It is infuriating to have to refer to page and line number, especially as page numbers are not even included. I have stated line numbers and I leave it up to the authors to find the relevant page.

While the presented genome assembly and annotation appear to have been conducted sensibly, the presented methods are inadequate at present to enable a meaningful assessment of this. There is value in the presented genome assembly compared to the available P. euphratica assembly in that the presented draft is more contiguous, however this requires validation. The included evolutionary analyses are potentially interesting but are limited in scope, contain no population level data and are entire descriptive. Such analyses are also frequently problematic when based on a draft genome assembly, and this problem is exacerbated here by use of two draft assemblies, with no assessment or apparent consideration for how this may influence to results obtained. As the associated web resource is currently non-functional and of limited scope (for example, there appears to be no link to P. trichocarpa or other species), the raft assembly is of limited potential use to the wider community.

L5-7. It is absolutely not true that little is known about the evolutionary genomics of Populus species. There is an existing body of literature relating to this and the evolutionary genomics and population genetics of various Populus species have been the focus of many past and recent studies. Of note, and also of relevance to the following sentence, the authors should be aware of the recent paper discussing the pan genome of P. nigra (https://doi.org/10.1093/molbev/msw161). There are also extensive genomics resources available for Populus. In short, this is a completely inadequate coverage of the extensive Populus literature that subsequently creates a false impression of novelty for the presented resource.

L14. What is your evidence for the statement that these two genomes do not cover the diversity observed? If this is an observation then state the source of this.

L15 Please include the authority for at first use of the species.

L26. Habitats are not specialised. Species living within habitats may evolve specialised adaptations to survive in those habitats.

L45 Unexpected to whom? There is nothing unexpected about the large number of variants identified,

with similar results reported for other Populus species.

The entire Data description section is wholly inadequate. Methods are not detailed, software versions and parameters used are not given. As such it would be completely impossible to recreate the presented work. For example, my group has experience using the Platanus assembler with Illumina data from highly heterozygous species and we would love to know how you ran the assembly to get such high contiguity.

Please give proper details about how the DNA was extracted, the kit versions used for the Illumina sequencing (this matters as different kit versions have different GC bias) and how the large-insert libraries were constructed.

L9. More detail is needed to understand how contaminated reads were removed. How were these classified? Was there any subsequent checking of the assembly to remove contaminant contigs?

L11. Be consistent in the terminology used for cleaned reads. They are also referred to as qualified reads, which I would suggest is not the most suitable or standard term.

The use of kmer based methods to estimate genome size is notoriously error prone. It would have been prefereable to see a range of kmers used to indicate likely error in genome size estimation. Even better would have been wet lab confirmation of genome size using flow cytometry. At the least, the use of the selected kmer size should be justified. It is also rather strange that the only parameters stated for any software are for KmerFreq_AR.

There is no attempt to validate or assess the quality of the assembly. This could have been done using one of the available tools (such as FRC analysis methods), for example.

L2. CEGMA and BUSCO are redundant and only one should be used. As detailed in the BUSCO publication, this is a replacement for CEGMA, with CEGMA no longer being under active development. I would also argue that a far more comprehensive analysis of gene space completeness could have been performed using the two available Populus genome annotations, as BUSCO is still rather limited. There are similarly more comprehensive such datasets available based on the PLAZA genome resource from Yves Van de Peer's group, where largely single copy genes have been identified across plant species. Please also refer to gene space completeness rather than genome completeness, as assessment of genes does not indicate how complete the entire assembly is.

L19-23. This could have been analysed more comprehensively. For example, it would have been highly informative to see an assessment of how many split haplotypes are represented in the assembly. This also has important implications for interpreting the presented gene family and synteny analyses.

L27. To state that GATK was used and to give no further details of how is, to be frank, ridiculous. The resulting heterozygosity estimate is entirely dependent on the settings used to run GATK so it is

impossible to form an opinion of whether this value has any meaning or not as presented. For example, how do the settings used compare to those used for the equivalent analysis in P. euphratica? Does this comparison hold any value whatsoever? To be meaningful, the extent of haplotype splitting in both assemblies would need to be assessed and taken into consideration as this has massive implications for SNP calling. As presented, I see no biological value in the stated comparison.

Gene annotation. I would have liked to see the logic of the presented annotation presented. For example, why was PASA used rather than using the available RNASeq support as input to Augustus? These various support evidence types are anyway then integrated using EVidenceModeler. Similarly, I would like ot know to justification for selecting the species used for protein support. Some of these genome are not high quality. There is also some confusing method description in this part. For example at L19 I do not follow the description of how homologous genome sequences were aligned. Why were partial genes and small genes removed and was any assessment performed of whether these may represent genes that are fragmented within the assembly?

The presented evolutionary analysis has, I believe, some important limitations and caveats. The most serious of these is the use of two draft, fragmented genome assemblies - which is particularly the case for P. euphractica. Draft assemblies are notoriously problematic for gene family analyses and for inference of contracted or expanded gene familes, for example. I would also have liked to see some comparison to other Populus spcies. For example, are the gene identified as rapidly evolving specific to the comparison of the two species considered or do these represent genes with generally relaxed selection constraint in common across a broader range of species? This section (and the subsequent gene family analyses) is also far too speculative and descriptive yet is the stated novelty of the presented resource.

L31 I have struggled to find this information at TimeTree for the two Populus species. Is there really fossil evidence to calibrate the divergence time for these two species specifically?

Availability of the genome: I have tried a number of times to visit the detailed web resource. On all occasions the genome and synteny browsers were non-functional. It also appears that only P. euphractica is available at the included BLAST tool. As such, the web resource appears to be of no value or use at present.

Many of the legends and details in the supplementary material are not adequate. For example, what is TRF? It is not clear whether any filter was applied for minimum contig/scaffold size when N50 etc statistics were calculated. Please ensure that all relevant details are given and that all abbreviations are defined.

Level of Interest

Please indicate how interesting you found the manuscript: An article of limited interest

Quality of Written English

Please indicate the quality of language in the manuscript: Acceptable

Declaration of Competing Interests

Please complete a declaration of competing interests, considering the following questions:

- Have you in the past five years received reimbursements, fees, funding, or salary from an organisation that may in any way gain or lose financially from the publication of this manuscript, either now or in the future?
- Do you hold any stocks or shares in an organisation that may in any way gain or lose financially from the publication of this manuscript, either now or in the future?
- Do you hold or are you currently applying for any patents relating to the content of the manuscript?
- Have you received reimbursements, fees, funding, or salary from an organization that holds or has applied for patents relating to the content of the manuscript?
- Do you have any other financial competing interests?
- Do you have any non-financial competing interests in relation to this paper?

If you can answer no to all of the above, write 'I declare that I have no competing interests' below. If your reply is yes to any, please give details below.

I declare that I have no competing interests

I agree to the open peer review policy of the journal. I understand that my name will be included on my report to the authors and, if the manuscript is accepted for publication, my named report including any attachments I upload will be posted on the website along with the authors' responses. I agree for my report to be made available under an Open Access Creative Commons CC-BY license (http://creativecommons.org/licenses/by/4.0/). I understand that any comments which I do not wish to be included in my named report can be included as confidential comments to the editors, which will not be published.

I agree to the open peer review policy of the journal
