# Supplementary material for: The draft genome sequence of a desert tree Populus pruinosa
Source: Gigascience. 2017 Aug 8;6(9):1–7. doi: 10.1093/gigascience/gix075 (PMC5603765; doi:10.1093/gigascience/gix075)
Supplement: Reviewer-2-Report-(Revision-1).pdf [file gix075_Reviewer-2-Report-(Revision-1).pdf]

Reviewer Report

Title: The draft genome sequence of a desert tree Populus pruinosa

Version: Revision 1 **Date:** 5/25/2017

Reviewer name: Nathaniel Street

Reviewer Comments to Author:

The authors have improved the original manuscript and have addressed a number of my original concerns. However, some do remain. That being said, I am satisfied that the presented work is of sufficient scientific quality.While it is good that the genome assembly and annotation is now made available, simply placing the fasta files on a FTP site does little service to the community. As the main benefit of the presented work is the potential for comparative analyses this is a great shame as the assembly is effectively of little utility to the community. I would strongly argue that the assembly and annotation should be placed at a central sequence resource such as NCBI or a Populus community resource.Unfortunately I have found reading the author responses a rather frustrating experience. There are no references to line numbers to help the reviewers locate the changed parts of the manuscript and changes have not been indicated (for example using coloured text). Many of the original concerns were not addressed, simply being replied to with "We have removed this" and those removed components were what actually offered some of the most potentially interesting biology. As such the revised manuscript is now very much a data release note, although the increased focus and removal of speculative or over-extrapolated hypotheses has benefited the present manuscript. As a general comment and suggestion to the authors when writing future responses to reviewers, I at least find it far more useful to be directed to the relevant changes and to be provided with some discussion in response to comments from the reviewers.Unfortunately there are still problems in the methods section - including the fact that readers are not directed to the relevant locations within the pipeline document provided on the GigaScience FTP site. For example (I am not detailing all similar such cases)L66 What is 'the CTAB method'? I know what it is personally, but this is not how to write a proper methods section. The CTAB method is based on an original publication and there are many subsequent variations to that original method.L67 Which Illumina protocol and what kit version?L75 I certainly would not be able to replicate this mate pair protocol given the provided detailsI appreciate that the online, main text version may need to be brief, but please then refer to a where full method details are provided.L99 A two year old plant is arguable no longer a seedling. How were these plants grown? How were the samples obtained?L146 What other assemblies and better in what way? Presumably not better than the P. trichocarpa assembly, so which other assemblies are being used for this comparison?L152 20X is actually not so deep if this relates to the two collapsed haplotypes, as this represents only 10X per haplotype.

Level of Interest

Please indicate how interesting you found the manuscript: An article of limited interest

Quality of Written English

Please indicate the quality of language in the manuscript: Acceptable

Declaration of Competing Interests

Please complete a declaration of competing interests, considering the following questions:

- Have you in the past five years received reimbursements, fees, funding, or salary from an organisation that may in any way gain or lose financially from the publication of this manuscript, either now or in the future?
- Do you hold any stocks or shares in an organisation that may in any way gain or lose financially from the publication of this manuscript, either now or in the future?
- Do you hold or are you currently applying for any patents relating to the content of the manuscript?
- Have you received reimbursements, fees, funding, or salary from an organization that holds or has applied for patents relating to the content of the manuscript?
- Do you have any other financial competing interests?
- Do you have any non-financial competing interests in relation to this paper?

If you can answer no to all of the above, write 'I declare that I have no competing interests' below. If your reply is yes to any, please give details below.

I declare that I have no competing interests

I agree to the open peer review policy of the journal. I understand that my name will be included on my report to the authors and, if the manuscript is accepted for publication, my named report including any attachments I upload will be posted on the website along with the authors' responses. I agree for my report to be made available under an Open Access Creative Commons CC-BY license (http://creativecommons.org/licenses/by/4.0/). I understand that any comments which I do not wish to be included in my named report can be included as confidential comments to the editors, which will not be published.

I agree to the open peer review policy of the journal
