# Supplementary material for: The draft genome sequence of a desert tree Populus pruinosa
Source: Gigascience. 2017 Aug 8;6(9):1–7. doi: 10.1093/gigascience/gix075 (PMC5603765; doi:10.1093/gigascience/gix075)
Supplement: Reviewer-3-Report-(Original-Submission).pdf [file gix075_Reviewer-3-Report-(Original-Submission).pdf]

Reviewer Report

Title: The draft genome sequence of a desert tree Populus pruinosa

Version: Original Submission **Date:** 2/12/2017

Reviewer name: John Mackay

Reviewer Comments to Author:

The Data note presents the genome assembly of Populus pruinosa - a poplar tree species that is adapted to the deserts of western China and neighbouring regions. It is the third genome of the Populus genus to be sequenced, and others are being developed. Poplars are important forest tree species from ecological and economic standpoints throughout the Northern hemisphere; therefore, the development of genome resources for poplars will have wide ranging impacts. The development of several genome sequences enables comparative and evolutionary studies and, the analysis of a desert-adapted species helps to develop knowledge that is relevant for adaptation to climate change and for combating desertification.This note describes the sequencing, assembly, annotation and evolutionary analysis of the genome. The methods are appropriate and the analyses are extensive. The data metrics indicate that the assembly is of high quality. The evolutionary analyses indicate that this resource is likely to lead to valuable developments in the understanding of adaptation and evolution in plants.The outcomes of several analyses are presented in the supplemental materials but why not include a composite figure presenting the key findings in the main paper? I feel it would be off interest to readers and strengthen the paper.I verified that the data have been deposited at the Short read archive for 7 Illumina libraries. I tried searching the project website (Salinity Tolerant Poplar Database) for information on the assembly. I did find a link but I was unsuccessful at downloading. Unfortunately I attempted this over Wifi which may be the problem. Could the authors give more detail on what is available and in what format? Could the authors also post an update on the website describing the genome assembly and its release?In the section 'Evolutionary analyses', the last sentence of the 1st paragraph states that the functional categories are probably related to the differences in the adaptations... Please explain the basis for the statement / hypothesis.In the next paragraph in the same sectin, the authors wrote that they identified 6,925 genes that are specific to P. pruinosa, which seems like a large number given what is known about gene conservation in plants. The authors should explain on what basis is the number obtained. Is the result relative to other poplars or relative to all plants?In the last sentence of the closing paragraph, the authors wrote that pan-analyses will be necessary in poplar but they do not explain why they believe this to be the case nor what it will achieve. I do not dispute the potential interest of the idea but I do feel it needs at least some explanation.

Level of Interest

Please indicate how interesting you found the manuscript: An article of importance in its field

Quality of Written English

Please indicate the quality of language in the manuscript: Acceptable

Declaration of Competing Interests

Please complete a declaration of competing interests, considering the following questions:

- Have you in the past five years received reimbursements, fees, funding, or salary from an organisation that may in any way gain or lose financially from the publication of this manuscript, either now or in the future?
- Do you hold any stocks or shares in an organisation that may in any way gain or lose financially from the publication of this manuscript, either now or in the future?
- Do you hold or are you currently applying for any patents relating to the content of the manuscript?
- Have you received reimbursements, fees, funding, or salary from an organization that holds or has applied for patents relating to the content of the manuscript?
- Do you have any other financial competing interests?
- Do you have any non-financial competing interests in relation to this paper?

If you can answer no to all of the above, write 'I declare that I have no competing interests' below. If your reply is yes to any, please give details below.

I declare that I have no competing interests

I agree to the open peer review policy of the journal. I understand that my name will be included on my report to the authors and, if the manuscript is accepted for publication, my named report including any attachments I upload will be posted on the website along with the authors' responses. I agree for my report to be made available under an Open Access Creative Commons CC-BY license (http://creativecommons.org/licenses/by/4.0/). I understand that any comments which I do not wish to be included in my named report can be included as confidential comments to the editors, which will not be published.

I agree to the open peer review policy of the journal
